# NT-seq: a chemical-based sequencing method for genomic methylome profiling

**DOI:** 10.1186/s13059-022-02689-9

**Published:** 2022-05-30

**Authors:** Xuwen Li, Shiyuan Guo, Yan Cui, Zijian Zhang, Xinlong Luo, Margarita T. Angelova, Laura F. Landweber, Yinsheng Wang, Tao P. Wu

**Affiliations:** 1 Department of Molecular and Human Genetics, Baylor College of Medicine, Houston, TX USA; 2 Genetics, Genomics, and Bioinformatics Graduate Program, University of California Riverside, Riverside, CA USA; 3 Departments of Biochemistry and Molecular Biophysics and Biological Sciences, Columbia University, New York, NY USA; 4 Department of Chemistry, University of California Riverside, Riverside, CA USA; 5 Huffington Center on Aging, Baylor College of Medicine, Houston, TX USA; 6 Dan L Duncan Comprehensive Cancer Center, Baylor College of Medicine, Houston, TX USA

**Keywords:** DNA methylation, Next-generation sequencing, Whole-genome epigenetic profiling

## Abstract

DNA methylation plays vital roles in both prokaryotes and eukaryotes. There are three forms of DNA methylation in prokaryotes: *N*^6^-methyladenine (6mA), *N*^4^-methylcytosine (4mC), and 5-methylcytosine (5mC). Although many sequencing methods have been developed to sequence specific types of methylation, few technologies can be used for efficiently mapping multiple types of methylation. Here, we present NT-seq for mapping all three types of methylation simultaneously. NT-seq reliably detects all known methylation motifs in two bacterial genomes and can be used for identifying de novo methylation motifs. NT-seq provides a simple and efficient solution for detecting multiple types of DNA methylation.

**Supplementary Information:**

The online version contains supplementary material available at 10.1186/s13059-022-02689-9.

## Background

Although epigenetic regulation has been reported in all domains of life, most studies focused on eukaryotes. However, mounting evidence for the crucial function of epigenetic regulatory pathways in prokaryotes has been reported. Three forms of DNA methylation, *N*^6^-methyladenine (6mA), *N*^4^-methylcytosine (4mC), and 5-methylcytosine (5mC), are prevalent and play essential roles in viral defense [1], mismatch repair [2], gene regulation [3, 4], and pathogenesis [4, 5] in prokaryotes. DNA methylation occurs in a motif-dependent manner in bacteria, and methylation motifs vary among different bacterial strains [6]. While emerging evidence has shown the functional role of bacterial methylation in transcriptional regulation, how DNA methylation and methyltransferases orchestrate the gene expression to determine the phenotype is still elusive [7]. One of the major challenges is the lack of efficient and straightforward methods for comprehensive genomic methylome profiling.

Most of the next-generation genomic sequencing (NGS) methods for DNA methylation mapping have been developed for 5mC, such as bisulfite sequencing [8], but 6mA is the most prevalent form of methylation in prokaryotes [9]. Multiple antibody-based or enzyme-based approaches have been developed [10–12], yet these methods are either complicated [10], low resolution [11], or restricted to particular enzyme-cutting motifs [12]. While the 3rd-generation single-molecule real-time sequencing (SMRT-seq) has been utilized to detect DNA methylation motifs in bacterial genomes [13, 14], the current SMRT-seq lacks open-source bioinformatic tools or independent methods that could cross-validate the results. Moreover, although SMRT-seq has been widely used to detect 6mA and 4mC in bacterial genomes, recent results from 4mC-TAB-seq [15] and mass spectrometry [16] indicated that SMRT-seq might overestimate 4mC in bacterial genomes. The Oxford Nanopore sequencing has also shown the ability to detect multiple types of DNA methylation in bacteria [17, 18], but the signal of Nanopore sequencing in detecting bacterial DNA methylation, especially the 6mA, is still noisy, and the machine-learning analysis methods need more training datasets [17]. Furthermore, since SMRT-seq and Nanopore sequencing can only detect methylation from unamplified genomic DNA, the required amount of input DNA is the limitation for applying single-molecule methods on restricted clinical samples. Therefore, to help fully understand microbial epigenomics, we must develop an efficient chemical-based NGS strategy to detect all three types of DNA methylation (whole methylome profiling).

DNA base deamination is a well-known chemical strategy to detect DNA methylation; for example, bisulfite sequencing, in which unmethylated cytosine is efficiently deaminated and converted to thymine during PCR amplification, while the modified cytosines such as 5mC and 5hmC are not converted [8]. For adenine methylation, although it was reported more than 60 years ago [19, 20], such a condition that could be applied for sequencing was not clarified until recently. Deamination induced by nitrous acid has been shown only to deaminate unmethylated adenine but not 6mA, which was utilized to develop nitrite sequencing [21] and NOseq [22] for DNA 6mA or RNA m6A detection in oligos or targeted sequencing settings. As far as we know, nitrite treatment has not been applied for genomic methylome profiling because generating the genomic sequencing library in such conditions and following bioinformatic analysis are still challenging [23].

Based on the previous studies, we developed NT-seq (nitrite treatment followed by next-generation sequencing), a sequencing method for detecting multiple types of DNA methylation genome-wide. We demonstrate that NT-seq can detect not only 6mA but also 4mC and 5mC. NT-seq can identify methylation motifs of all three types of methylation in *Escherichia coli* and *Helicobacter pylori* genomes. We also show that NT-seq can be used for methylation motif de novo discovery in a microbial community standard sample. Thus, NT-seq provides an efficient, cost-effective, and high-resolution method for methylation motif detection in both single bacterial species and metagenomic settings. Of particular note, 6mA has also been reported in lower eukaryotes and mammals [24–27]. The 6mA methylated DNA immunoprecipitation followed by sequencing (6mA DIP-seq) is the primary approach for profiling 6mA in eukaryotic genomes, but the specificity has been debated [28, 29]. Since our method can efficiently recognize 6mA, coupled with DIP-Seq, we present that DIP-NT-seq can detect 6mA at single-base resolution with high fidelity and eliminate the false-positive 6mA sites in DIP-seq. Thus, this method can be used independently or coupled with other protocols for methylome profiling, which will pave the way for DNA modification studies in different contexts.

## Results

### Experimental design of NT-seq

**Fig. 1:**
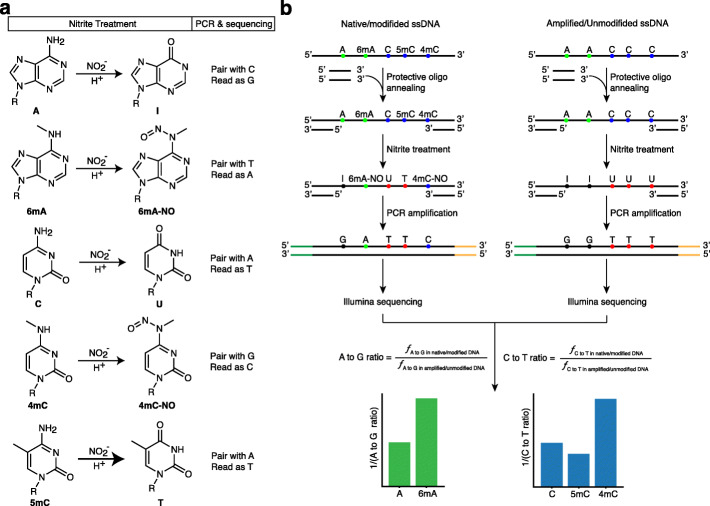
Principle and workflow of NT-seq. **a** Schematic illustration of nitrite treatment. Nitrite treatment induces deamination of adenine, cytosine, and 5mC at different frequencies, producing inosine, uracil, and thymine, respectively. Meanwhile, nitrite treatment nitrosylates 6mA and 4mC, producing nitrosylated 6mA (6mA-NO) and nitrosylated 4mC (4mC-NO). During PCR amplification and sequencing, base pairing and reading for each product are labeled on the right column. **b** The workflow of NT-seq. Single-stranded DNA is first annealed with protective oligos to protect PCR primer regions. Annealed DNA is treated with nitrite and then amplified to construct the sequencing library. Sequencing data from native DNA and PCR control are used to calculate the A to G or C to T mutation ratio and to call methylation

Nitrite treatment has been reported to deaminate adenine (A), cytosine (C), and guanine (G) for decades [30, 31]. The deamination of A or C changes the bases and is read by polymerases as G or T, respectively, in PCR amplification (Fig. 1a) [30, 31]. As the methyl groups of 6mA and 4mC are located at the amino groups of adenine and cytosine, 6mA and 4mC can block the deamination of adenine and cytosine under nitrite conditions. Recently, liquid chromatography mass spectrometry (LC-MS) results showed that *N*^6^-methyladenosine (m^6^A) was converted to *N*^6^-nitroso-m6A (m^6^A-NO) but not deaminated inosine by nitrite treatment [21, 22]. While the nitrite treatment has been adapted to detect DNA 6mA and RNA m^6^A, it has been only tested in oligos or targeted RNA locus as a proof-of-concept for methylation sequencing [21, 22]. Moreover, it can also be used to distinguish 5mC from cytosine because the deamination rate of 5mC in nitrite treatment has been reported to be up to 4.5-fold higher than cytosine [32] (Fig. 1a). However, whether this approach could be applied for whole-genome sequencing (WGS) with genomic DNA libraries remains unknown. Here, we hypothesize that the DNA 4mC, like the 6mA, can also be detected with nitrite treatment, and we can build the NGS sequencing library for NGS sequencing to define the whole methylome profiling (Fig. 1a).

We first characterized the products formed from the reactions between nitrite and 2′-deoxyadenosine/2′-deoxycytidine by HPLC separation followed by mass spectrometric analyses. We found that the two nucleosides can indeed be deaminated upon subjected to nitrite treatment (Additional file 1: Fig. S1–S4). More importantly, when subjecting 6mA and 4mC to the same nitrite treatment conditions, 6mA-NO and 4mC-NO were the dominant products and there were minimal side products formed from nitrite treatment (Additional file 1: Fig. S5–S8). Additionally, we performed time-dependent nitrite treatment to investigate the reaction dynamics of these four nucleosides. Consistent with published m^6^A results [21], we found that 6mA and 4mC are converted to 6mA-NO and 4mC-NO more rapidly than the deamination of their unmethylated counterparts (Additional file 1: Fig. S9).

We then designed an experimental workflow to investigate whether nitrite treatment can be used to develop a sequencing method to simultaneously detect genome-wide all three types of methylation (Fig. 1b). In the workflow, we hybridized two protective oligos, which is reverse complementary to the primer regions of the single-stranded DNA (ssDNA) since Watson-Crick base pairing has been shown to protect DNA bases from deamination [33]. The quantitative PCR (qPCR) results showed that the protected DNA could be more efficiently amplified than unprotected DNA by decreasing the cycle threshold (*Ct*) value up to 7.5 (Additional file 2: Table S1). Since the deamination by nitrite treatment is completed through nitrite-mediated diazotization followed by hydrolysis which requires acidic conditions and relatively high temperature, the deamination efficiency is positively correlated to acid concentration, incubation temperature, and treatment duration [21]. However, increasing these conditions such as treatment duration can also result in an increased level of DNA degradation (Additional file 1: Fig. S10). Therefore, it is crucial to optimize the treatment condition to achieve a high deamination rate while preserving enough DNA for library preparation when applying it to genomic DNA. Therefore, we treated six pmol (~250 ng) protected DNA oligos with different concentrations of acetic acid, different temperatures, and different durations of treatment and performed qPCR to estimate the amount of remaining amplifiable fragments. Considering Ct around 15 as the required amount of amplifiable DNA for downstream library preparation, we determined 2.3% acetic acid, 1 M sodium nitrite (pH = 4.187), and incubation at 37 °C as our optimal condition (Additional file 3: Table S2). We also evaluated the damage level caused by nitrite treatment using 293T genomic DNA and found that nitrite-treated DNA is less fragmented compared to bisulfite-treated DNA. However, the DNA degradation from nitrite treatment is more severe than bisulfite treatment (Additional file 1: Fig. S10).

To enable methylation detection in genomic DNA, it is also essential to efficiently align NT-seq reads to the reference genome. Therefore, we developed an NT-seq analysis pipeline that can tolerate all deamination-elicited base substitutions (Additional file 1: Fig. S11). Since the base deamination will only cause transition mutations (A to G, C to T, or G to A) but not transversion mutations, we degenerate A/C/G/T bases in both reads and reference to purine/pyrimidine bases. Using whole-genome sequencing data from *E. coli* MG1655 as a mock dataset, we artificially introduced A to G, C to T, or G to A change to mimic the base changes after nitrite treatment. The unique alignment rate is very similar to the original unchanged reads, indicating the NT-seq analysis pipeline can tolerate all possible base changes introduced by nitrite treatment (Additional file 1: Fig. S11).

### Detection of 6mA, 4mC, and 5mC in oligonucleotides using NT-seq

**Fig. 2:**
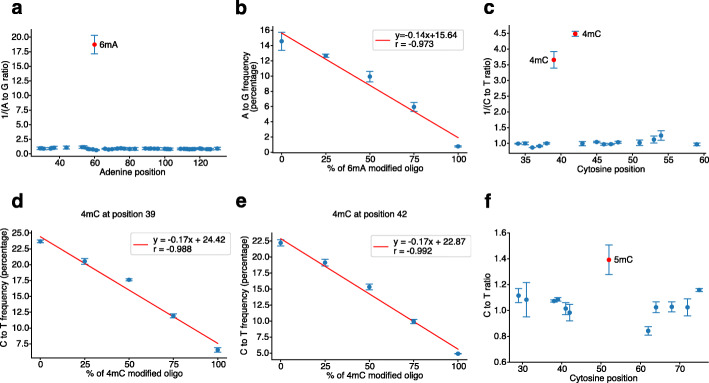
NT-seq detects both adenine and cytosine methylation in oligonucleotides. **a** The inverse of A to G ratio at adenine sites between unmodified control and 6mA modified oligo. **b** Linear regression between A to G frequency at 6mA site and the percentage of 6mA modified oligo. **c** The inverse of C to T ratio at cytosine sites between unmodified oligo and oligo modified by BamHI methyltransferase. **d** Linear regression between C to T frequency at 4mC position 39 and the percentage of 4mC modified oligo. **e** Linear regression between C to T frequency at 4mC position 42 and the percentage of 4mC modified oligo. **f** C to T ratio at cytosine sites between unmodified control and 5mC modified oligo. Modified adenine or cytosine sites are labeled in red. Adenine or cytosine sites inside the primer regions are not included. Dots represent the mean and error bars represent standard deviation. All samples were replicated three times. One replicate for 25%, 50%, and 100% of 4mC modified oligo samples was not used due to library prep and sequencing depth issues

To investigate the feasibility of NT-seq for DNA 6mA methylation detection, we performed pilot experiments with 6mA modified oligo and unmodified oligo. Consistent with previous work [21], we found that at the 6mA position, the A to G ratio (the ratio between A to G frequency in modified/native sample and A to G frequency in unmodified/amplified sample) is about 18-fold lower than that in other adenine positions (Fig. 2a). We further performed NT-seq on oligos mixed with different percentages of 6mA modified oligo and found that the normalized A to G frequency at the 6mA position is linearly correlated to the 6mA percentage (*r* = −0.968, Fig. 2b), indicating that the NT-seq can quantify the 6mA frequency precisely.

We then used BamHI methyltransferase to methylate double-strand DNA oligo with GGATCC motif for 4mC modification, as the 4mC modified DNA oligo is not commercially available. The BamHI restriction enzyme was used to cleave unmethylated DNA oligo before nitrite treatment (Additional file 1: Fig. S12). The NT-seq result showed that C to T ratio at the BamHI methylated sites is about 4-fold lower than other cytosine positions (Fig. 2c). Additionally, we performed NT-seq on oligos mixed with different percentages of 4mC modified oligo and demonstrated the C to T ratio at both 4mC positions is linearly correlated to 4mC percentage (*r* = −0.988 for position 39 and −0.992 for position 42), indicating that NT-seq can also quantify 4mC frequency (Fig. 2d, e). These results demonstrated that NT-seq indeed could detect 4mC and 6mA in parallel. Of note, the fold change of 4mC oligo is lower than 6mA oligo, which is likely caused by a small proportion of hemimethylated/unmethylated 4mC oligo that escaped from restriction enzyme cleavage.

As for detecting 5mC using NT-seq, we performed NT-seq on 5mC modified and unmodified oligos. The C to T ratio at the 5mC position is about 40% higher than other cytosine positions (Fig. 2f), consistent with a previous study showing that 5mC is easier to deaminate than C [32]. We also performed NT-seq on oligo mixture with different percentages of 5mC oligo and found that the C to T frequency is also linearly correlated to the percentage of 5mC (*r* = 0.972, Additional file 1: Fig. S12). This result further demonstrated that NT-seq could also detect 5mC quantitatively. Notably, unlike bisulfite sequencing, the impact of 4mC and 5mC is the opposite during nitrite treatment, making NT-seq capable of distinguishing 4mC and 5mC. Taken together, we demonstrated that NT-seq could detect all three types of DNA methylation in DNA oligos.

### Detection of methylation motifs in bacteria by NT-seq

**Fig. 3:**
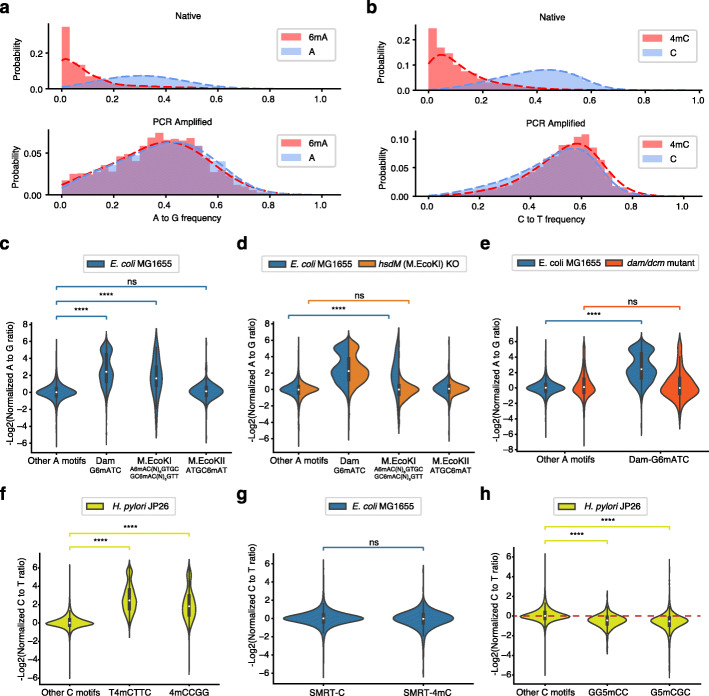
NT-seq simultaneously detects three types of methylation motifs in bacteria. **a** A to G frequency at known 6mA sites (G6mATC, A6mACN_6_GTGC, and GC6mACN_6_GTT) and unmethylated A sites in *E. coli* MG1655 genome from native and PCR amplified DNA. **b** C to T frequency at known 4mC sites (T4mCTTC and 4mCCGG) and unmethylated C sites in *H. pylori* JP26 genome from native and PCR amplified DNA. **c–e** Negative log normalized A to G ratio of different 6mA motifs in *E. coli* WT strain MG1655 (**a**), *hsdM* deleted strain (**b**), and *dam/dcm/hsdR* mutated strain (**c**). 6mA position was underlined. **f** Negative log normalized C to T ratio of different 4mC motifs in *H. pylori* JP26. **g** Negative log normalized C to T ratio of 4mC sites identified by SMRT-seq in *E. coli* strain MG1655. **h** Negative log normalized C to T ratio of different 5mC motifs in *H. pylori* JP26. Only motifs with sequencing depth larger than 25× were considered for violin plots. Statistic test were performed by two-sided Mann-Whitney-Wilcoxon (MWW) test with Bonferroni correction (ns: *P* > 1.0e-3; ****: *P* ≤ 1.0e−6)

To determine the capability of NT-seq in detecting three types of methylation motif in genomic DNA, we applied NT-seq to the *E. coli* MG1655 genome. Firstly, we found that A to G frequency at known 6mA sites (Dam (G6mATC) and M.EcoKI (A6mACN_6_GTGC and GC6mACN_6_GTT) motifs) was significantly decreased compared to unmethylated adenine sites, while no difference was observed after PCR amplification (Fig. 3a). Thus, the A to G ratio at Dam and M.EcoKI motifs was significantly decreased compared to unmethylated adenine positions (Fig. 3c). The M.EcoKII motif was used as a negative control because the methyltransferase M.EcoKII is known not to be expressed under standard laboratory conditions [34]. As expected, the A to G ratio at the M.EcoKII motif is not different from other unmethylated adenine sites (Fig. 3c). To further demonstrate that NT-seq detects bona fide 6mA motifs, we applied NT-seq to *hsdM* (the gene encoding M.EcoKI protein) KO strain and *dam/dcm* mutated strain. In contrast to the WT *E. coli* strain, the M.EcoKI motif in the *hsdM* KO strain shows no difference in A to G ratio from other unmethylated adenine sites (Fig. 3d). Similarly, the A to G ratio difference between the Dam motif and unmethylated adenine is also lost in the *dam/dcm* mutated strain (Fig. 3e).

Next, we performed NT-seq on the *H. pylori* JP26 genome, which contains two known 4mC motifs: 4mCCGG and T4mCTTC [17]. Consistent with 6mA results, the C to T frequency at these two 4mC motifs was decreased compared to unmethylated cytosine positions, while no difference was observed after PCR amplification (Fig. 3b). The C to T ratio at these two motifs decreased around 4-fold compared to unmethylated cytosine positions (Fig. 3f). As there is no known 4mC motif in *E. coli* MG1655, we analyzed C to T ratio at 4mC sites identified by SMRT-seq [35]. Consistent with the previous report, the C to T ratio at these sites showed no difference from other cytosine sites (Fig. 3g), indicating NT-seq can only detect true 4mC-induced difference. In contrast, the A to G ratio at 6mA sites identified by SMRT-seq was decreased 4-fold on average (Additional file 1: Fig. S13). These results are consistent with the previous report [15, 16] that SMRT-seq may overestimate the total level of 4mC or falsely detect some 4mC motifs in the bacterial genomes.

We compared C to T ratio at two known 5mC motifs in the *H. pylori* genome to examine whether NT-seq also detects 5mC motifs. Consistent with 5mC oligo results, the C to T ratio at these two known 5mC motifs is significantly increased compared to unmethylated cytosine sites (Fig. 3h). Similarly, the Dcm motif in *E. coli* also shows a significantly increased C to T ratio between native and PCR samples (Additional file 1: Fig. S13). The increase in C to T ratio is diminished in *dam/dcm* mutant, further indicating that the 5mC can be detected by NT-seq (Additional file 1: Fig. S13). Overall, we demonstrated NT-seq could be used to simultaneously detect multiple types of DNA methylation motif in bacteria genomes.

### De novo discovery of methylation motifs in bacteria using NT-seq

**Fig. 4:**
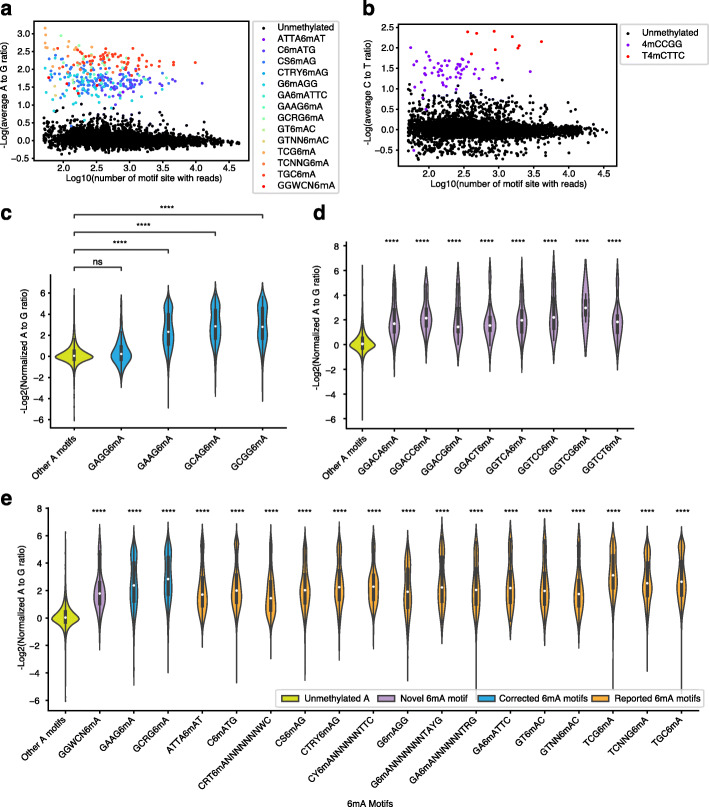
De novo discovery of methylation motifs in *H. pylori* genome using NT-seq. **a-b** Scatter plot of the median difference of −Log2FC between any 4mer-6mer A motif and the remaining A sites (**a**) and the median difference of -LogFC between any 4mer-6mer C motif and the remaining C sites (**b**). (Previously reported, corrected, and novel 6mA motifs are labeled in color). **c** Violin plot of −Log2(Normalized A to G ratio) for four sub motifs of previous reported GMRG6mA motif. **d** Violin plot of −Log2(Normalized A to G ratio) for eight sub motifs of GGWCN6mA motif. **e** Violin plot of −Log2(Normalized A to G ratio) for all previous reported and newly discovered 6mA motifs. Statistic test were performed by two-sided Mann-Whitney-Wilcoxon (MWW) test with Bonferroni correction (ns: *P* > 1.0e-3; ****: *P* ≤ 1.0e−6)

As we showed that NT-seq could detect known methylation motifs in bacteria, we further explored whether we could discover novel methylation motifs using NT-seq. We traversed all possible 4mer, 5mer, and 6mer adenine motifs in the *H. pylori* JP26 genome and found that the average A to G ratio of these motifs can be clearly divided into two groups (Additional file 1: Fig. S14). Most of the upper group are known 6mA motifs, and the lower group includes mainly unmethylated motifs with few exceptions. We further investigated these exceptions and found one sub-motif (GAGG6mA) of a previously reported motif (GMRG6mA) showed no difference in A to G ratio from other adenine motifs. In contrast, the other three sub motifs are significantly different from other adenine motifs (Fig. 4c). The same pattern was observed in SMRT-seq results (Additional file 1: Fig. S14), demonstrating that previous SMRT-seq workflow imprecisely combined GAAG6mA and GCRG6mA into GMRG6mA.

Moreover, we identified a novel 6mA motif–GGWCN6mA using NT-seq (Fig. 4d), which has not been identified by SMRT-seq in the previous study. We investigated the sub motifs of GGWCN6mA and found all sub motifs showed a significant decrease in A to G ratio compared to unmethylated motifs (Fig. 4d). A similar trend was also observed in the SMRT-seq IPD ratio quantification, demonstrating GGWCN6mA is an actual 6mA motif (Additional file 1: Fig. S14). Complete annotation of the 4-6mer 6mA motif scatter plot and the detailed distribution of the A to G ratio of all 6mA motifs in the *H. pylori* genome are shown in Fig. 4a and e. By contrast, we did the same analysis for cytosine motifs and failed to find novel 4mC or 5mC motifs in the *H. Pylori* JP26 genome, indicating there is likely no additional cytosine methylation motif other than previously reported 4mCCGG, T4mCTTC, G5mCGC, and GG5mCC (Fig. 4b, Additional file 1: Fig. S14).

These results demonstrated that NT-seq could not only validate reported methylation motifs but also identify novel methylation motifs in bacterial genomes. Therefore, NT-seq provides a simple “all-in-one” solution to accurately profile methylome in bacterial genomes, which will facilitate the discovery of unknown methylation motifs and epigenetic regulators in bacteria.

### Methylation motif identification in microbial community reference using NT-seq

**Fig 5:**
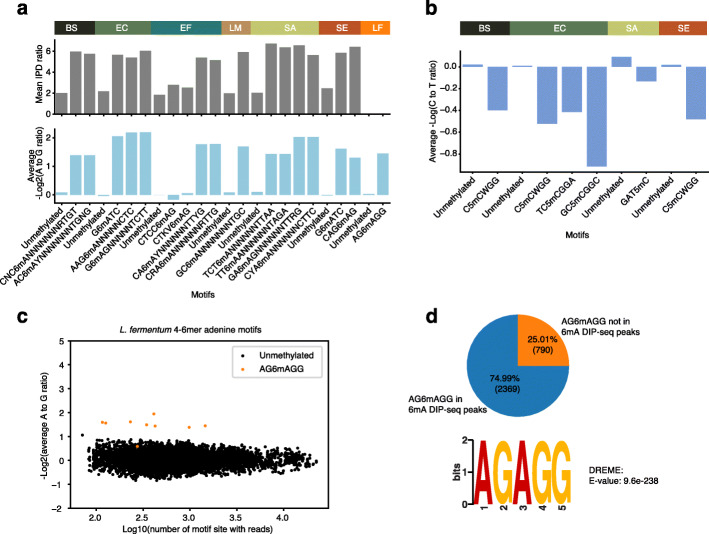
Methylation motifs detection in microbial community reference. **a** Mean IPD ratio from SMRT-seq and average −log2(A to G ratio) from NT-seq on unmethylated motifs and 6mA methylated motifs in microbial community reference. **b** Average −log2(C to T ratio) from NT-seq on unmethylated motifs and 4-6mer 5mC methylated motifs in microbial community reference. **c** Scatter plot of average −log2(A to G ratio) and log10(number of motif site with reads) for all 4-6mer adenine motifs in *L. fermentum* genome. **d** 6mA DIP-seq peaks overlap with the majority of AGAGG motif sites in the *L. fermentum* genome and AGAGG is the top enriched motif in 6mA DIP-seq peaks. (BS: *B. subtilis*, EC: *E. coli*, EF: *E. faecalis*, LM: *Listeria monocytogenes*, SA: *S. aureus*, SE: *S. enterica*, LF: *L. fermentum*)

Meta-epigenomic analysis based on SMRT-seq has recently revealed diverse DNA methylation in an environmental microbial community [36]. To determine whether NT-seq can be used to detect DNA methylation patterns in microbial community samples, we applied NT-seq on a commercial microbial community standard, including eight bacteria species and two fungi species. Since the composition of two fungi genomes is about six times lower than other bacterial genomes, and there is no reported methylation motif in both fungi species, we focused our 6mA analysis on eight bacteria species. We traversed all possible 4mer, 5mer, 6mer, and common type I RM bipartite methylated adenine motifs. We confirmed that all 6mA motifs with a high IPD ratio from the previous SMRT-seq [18] in seven bacteria strains are significantly different from unmethylated motifs in NT-seq (Fig. 5a, Additional file 1: Fig. S15, S16).

Interestingly, putative 6mA motifs (CTKV6mAG and CTCC6mAG in *E. faecalis*) with a small difference in IPD ratio from SMRT-seq showed no difference in NT-seq, indicating that these two motifs might not be 6mA methylated (Fig. 5a). Consistently, the current signals from Nanopore sequencing showed minor changes at any position of CTKVAG or CTCCAG [18]. For *Lactobacillus fermentum*, which has no available SMRT-seq data, a putative 6mA motif: AG6mAGG showed a similar extent of decrease in A to G ratio compared to known 6mA motifs in other species (Fig. 5a). A to G ratio distribution of most sub motifs of AG6mAGG displayed clear separation from unmethylated motifs, suggesting it is a true 6mA motif (Fig. 5c, Additional file 1: Fig. S15). A previous study using 6mA DIP-seq failed to detect any 6mA peaks in the *L. fermentum* genome [18]. Therefore, we reanalyzed their 6mA DIP-seq data and found we were able to identify 1557 6mA peaks by keeping all “duplication” reads at the exact location but failed to detect any peaks after default deduplication. The reads “duplication” here can be caused by excessive sequencing depth (15.7 million reads on average for a 1.9 million genome) rather than true PCR duplicates. Further investigation of identified 1557 6mA peaks revealed 75% of total AG6mAGG motifs intersected with 6mA peaks (Fig. 5d). Motif discovery analysis also identified AGAGG as the top significantly enriched motif inside these 6mA peaks (Fig. 5d). These results clearly demonstrated the AG6mAGG detected by NT-seq is indeed a true 6mA motif in the *L. fermentum* genome.

For cytosine methylation, we were not able to identify any 4mC methylation motif, indicating there is likely no 4mC motif in these bacteria genomes. We also analyzed available bisulfite sequencing for all eight bacterial species and identified six 4 to 6mer cytosine methylation motifs in *Bacillus subtilis*, *E. coli*, *Staphylococcus aureus*, and *Salmonella enterica* (Additional file 4: Table S3). The C to T ratios of NT-seq at these motifs were all higher than unmethylation motif controls (Fig. 5b), indicating that these cytosine methylation motifs are 5mC methylated but not 4mC methylated.

### Profiling 6mA at single-base resolution in E. coli and Oxytricha genome by DIP-NT-seq

**Fig 6:**
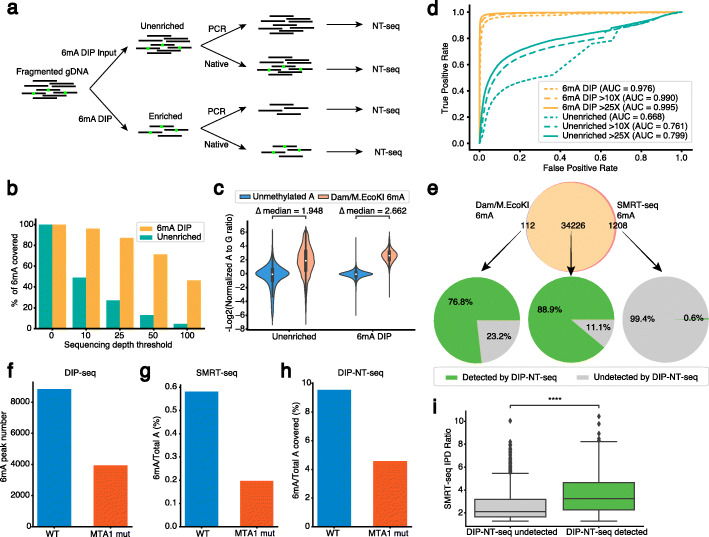
6mA profiling at single-base resolution in the bacterial and eukaryotic genomes. **a** Workflow of DNA 6mA immunoprecipitation followed by NT-seq (6mA DIP-NT-seq). 6mA modified DNA fragment is labeled in green. **b** Bar plot of percentage of 6mA sites passing filter by different sequencing depth threshold. 100% at threshold 0 means there is no filtering. **c** Violin plot shows 6mA DIP increases the A to G ratio difference between 6mA and A. **d** Receiver operating characteristic (ROC) curve shows that 6mA DIP significantly improves the performance of 6mA detection at single-base resolution. **e** Pie charts of DIP-NT-seq in 6mA sites correctly and incorrectly classified by SMRT-seq. Normalized A to G ratio threshold was determined by maximizing the F1 score. **f** 6mA DIP-seq peak number in WT and MTA1 mutant *Oxytricha* strain. **g** SMRT-seq 6mA percentage in WT and MTA1 mutant *Oxytricha* strain. **h** DIP-NT-seq 6mA percentage in WT and MTA1 mutant *Oxytricha* strain. **i** IPD ratio of DIP-NT-seq undetected and detected 6mA. Statistic tests were performed by two-sided Mann-Whitney-Wilcoxon (MWW) test with Bonferroni correction (****: *P* ≤ 1.0e−6)

DNA 6mA is the most common methylation in prokaryotes. Recently, 6mA has been identified in eukaryotes, including mammals, in which DNA 6mA methylation is motif independent. To evaluate the performance of NT-seq in detecting different types of DNA methylation at the single-base resolution, we generated a Receiver operating characteristic (ROC) curve for each methylation type in the *H. pylori* genome (Additional file 1: Fig. S17). Consistent with oligo results, the performance of NT-seq in detecting 6mA and 4mC at single-base resolution is similar (AUC = 0.934 for 6mA and 0.948 for 4mC at positions with more than 25× coverage). The performance is significantly decreased for 5mC detection (AUC = 0.832 at positions more than 25× coverage). To explore whether the performance of NT-seq in detecting methylation at single-base resolution can be further improved, we coupled 6mA DNA immunoprecipitation (6mA-DIP) with NT-seq and tested it in the *E. coli* genome (Fig. 6a). We sequenced both the unenriched whole-genome sample and 6mA-DIP-enriched sample to a near saturation depth as indicated by PCR duplication level (Additional file 5: Table S4). We found that without enrichment, NT-seq is unable to cover the whole set of the 6mA sites with high sequencing depth (25×, Fig. 6b). However, with 6mA-DIP enrichment, NT-seq can cover roughly 3-fold more 6mA sites at the same sequencing depth threshold (25×, Fig. 6b). This might be due to the DNA damage effect induced by nitrite treatment under acidic conditions. Previous studies showed that depurination in xanthine (generated from guanine deamination) is more rapid than other bases under acidic conditions [37, 38]. Additionally, xanthine was reported to induce polymerase arrest [37], which may lead to a PCR amplification bias for xanthine-free DNA fragments. Consistently, compared to untreated samples, nitrite treatment causes the final sequencing result to be more biased for G-poor regions (Additional file 1: Fig. S18). Therefore, 6mA-DIP can eliminate reads from unmethylated G-poor regions and concentrate sequencing reads to methylation loci. Additionally, 6mA-DIP also enriched 6mA, as shown by a significant decrease of A to G ratio at Dam/M.EcoKI motif in unenriched sample when compared to enriched sample (Fig. 6c). To comprehensively evaluate the performance of NT-seq in detecting 6mA sites, we generated the ROC curve of unenriched and DIP-enriched samples using Dam/M.EcoKI motif as the gold standard for 6mA positions. Consistently, DIP enrichment greatly improves the AUC scores at different sequencing depth thresholds (Fig. 6d). Similar results were also observed in the PR curve and AP scores (Additional file 1: Fig. S18).

By comparing DIP-NT-seq with the well-established SMRT-seq method on 6mA single-base detection, we found DIP-NT-seq and SMRT-seq results are consistent (Additional file 1: Fig. S18). Furthermore, using Dam/M.EcoKI-mediated 6mA as the ground truth, DIP-NT-seq can detect up to 76.8% of the false-negative sites from SMRT-seq and only 0.3% of false-positive sites from SMRT-seq, indicating that DIP-NT-seq was able to efficiently filter false-positive 6mA identified from SMRT-seq. In addition to SMRT-seq, we also compared DIP-NT-seq to a few other next-generation sequencing-based methods for *E. coli* 6mA detection, such as 6mA DIP-seq [18] and 6mACE-seq [10]. We showed that DIP-NT-seq can detect 11.7% more 6mA sites within the Dam (G6mATC) motif, indicating DIP-NT-seq can not only increase traditional DIP-seq to single-base resolution but also increase the sensitivity for 6mA detection (Additional file 1: Fig. S19). When comparing to 6mACE-seq, we found that DIP-NT-seq can detect 7% more 6mA sites but DIP-NT-seq generates more false-positive sites (Additional file 1: Fig. S19). Overall, F1 score indicates DIP-NT-seq is slightly improved compared to 6mACE-seq (0.843 for DIP-NT-seq and 0.833 for 6mACE-seq). It is also worth noting that the performance of NT-seq in identifying true 6mA can be improved using a higher sequencing depth threshold (25X, F1 score: 0.911; 50X, F1 score: 0.953) (Additional file 1: Fig. S18), indicating these incorrectly classified 6mA sites by NT-seq were likely caused by the low sequencing coverage at these sites, which can be solved by increasing the sequencing depth.

6mA has recently been described in various eukaryotic organisms, including unicellular eukaryotes [24, 39], metazoans [25–27], and plants [40, 41]. 6mA is generally more abundant in unicellular eukaryotes than metazoans. In *Oxytricha trifallax*, the abundance of 6mA is around 7000 ppm (parts per million dA), and MTA1c has been identified as a methyltransferase complex of 6mA [39]. Although the 6mA DIP-seq is the primary approach for profiling 6mA in eukaryotic genomes, the specificity of this antibody-based method has been debated [28, 29]. To determine whether NT-seq can improve the specificity and resolution of DIP-seq, we performed DIP-NT-seq in WT and MTA1 mutated *Oxytricha*. In agreement with 6mA DIP-seq and SMRT-seq results [39], 6mA level in MTA1 mutant is about 50% less than WT *Oxytricha* (Fig. 6f–h), suggesting that DIP-NT-seq can robustly detect 6mA changes at single-base resolution in eukaryotic genomes. The SMRT-seq IPD ratio of 6mA detected by DIP-NT-seq is significantly higher than 6mA undetected by DIP-NT-seq (Fig. 6i), indicating that 6mA sites detected by DIP-NT-seq can be cross-validated in SMRT-seq.

## Discussion

In this study, we developed NT-seq to map multiple types of DNA methylation genome-wide. We demonstrated that NT-seq could be used to detect all three major types of methylation motifs in bacterial genomes simultaneously. Notably, NT-seq can not only detect known methylation motifs but also be used to discover novel methylation motifs. We demonstrated that NT-seq could be used for de novo methylation motif discovery in single bacteria species and microbial communities. Furthermore, our results also indicate that coupling methyl DNA immunoprecipitation (DIP) and NT-seq can profile 6mA at single-base resolution in both prokaryotes and eukaryotes. We demonstrated that the performance of DIP-NT-seq in the *E. coli* genome is comparable to SMRT-seq. Moreover, we demonstrated that DIP-NT-seq could filter out the false-positive 6mA sites from DIP-seq or SMRT sequencing, which raised the concerns for 6mA detection in the field. Therefore, NT-seq improves the accuracy of 6mA detection and supply an independent method that can be used for cross-validation with SMRT-seq.

While 6mA has been identified in eukaryotes, including mammals [24–27], current 6mA genomic profiling in eukaryotes are mainly dependent on 6mA antibody-based DIP-seq. However, 6mA antibodies have recently been shown might generate non-specific signals when the abundance of 6mA is low [28, 29]. In fact, Wu TP. et al. have reported the 6mA DIP-seq sensitivity limitation in 2016. By coupling 6mA DIP and NT-seq, the non-6mA fragments (false-positive source) will not generate different A to G ratios than PCR control in nitrite treatment. Thus, NT-seq can improve the specificity and resolution of 6mA DIP-seq by filtering out false-positive signals.

Here, we would also like to discuss a few limitations of NT-seq. Firstly, the current nitrite treatment in NT-seq causes DNA damage, which causes trouble for library generation and makes us unable to completely deaminate A and C to reach a better performance (Fig. 3a, b; Additional file 1: Fig. S13). Secondly, we found that NT-seq reads are biased on G-poor regions (Additional file 1: Fig. S18), which may limit the application of NT-seq in GC-rich bacterial genomes, although we took advantage of annealing two protective oligos on the PCR primer regions before nitrite treatment to preserve amplifiable DNA fragments and can generate the NGS library of most of the genomic DNA. In the bacterial genomes we tested, *Pseudomonas aeruginosa* has the highest GC content (66.2%), and we did find a slightly noisier distribution of A to G ratio in different motifs, compared to other genomes with low GC content (Additional file 1: Fig. S15). Therefore, high GC content bacterial genome may require more input DNA to achieve identical performance than low GC content genomes. Lastly, the 5mC detection by NT-seq is less efficient than bisulfite sequencing. Thus, we recommend performing a shallow bisulfite sequencing to cross-validate the NT-seq-detected 5mC motifs for unknown genomes or microbial community samples. These limitations might be overcome by searching for a mild nitrite treatment condition to achieve near complete deamination rate while decrease the DNA degradation.

Collectively, NT-seq provides an efficient chemical-based method to reliably detect multiple types of DNA methylation in single bacteria, microbial communities, and eukaryotic genomes. Importantly, since NGS is much more affordable and easier to access than current third-generation sequencing platforms, NT-seq makes bacterial methylation analysis more accessible and cost-efficient for epigenetic researchers. Recently, Nanopore sequencing was shown to be able to detect multiple types of DNA methylation motifs in bacteria and microbiome. However, accurate methylation detection from Nanopore sequencing requires computational training with known methylation motifs sequencing in the different contexts, which are currently generated from the high-cost SMRT-seq. To aim for mammalian methylome profiling with Nanopore sequencing, more training datasets will be necessary for the success analysis model development. Recent developed computational tools like nanodisco [17] are still insufficient to reliably detect 6mA motifs in mouse gut microbiome samples (Additional file 1: Fig. S20), largely limited by the small training dataset (only 28 known 6mA motifs trained *versus* hundreds of thousands of possible 6mA motifs in microbial genomes). Since we have shown that NT-seq can reliably detect multiple types of DNA methylation motifs, NT-seq can be used to generate more accurate and large-scale training datasets with minimal cost and help to develop machine-learning-based computational tools for methylation detection from Nanopore and other single-molecule sequencing.

## Conclusions

We developed a method (NT-seq) to simultaneously map all three major types of DNA methylation in prokaryotic genomes. NT-seq allows accurate detection of methylation motifs in both single species and microbial community samples. Compared to SMRT-seq, NT-seq provides a cost-efficient solution for bacterial methylation mapping, which will boost the study of bacterial epigenetics. By coupling methyl DNA immunoprecipitation (DIP), we showed that NT-seq could accurately profile 6mA at single-base resolution in bacterial genomes, which could also be applied to eukaryotic genomes to help eliminating the non-specific signals in 6mA DIP-seq in eukaryotes. NT-Seq can cross-validate SMRT-Seq results and generate more training datasets for developing machine-learning tools for methylation analysis. This method paves the way for further epigenetic study on genomic DNA 6mA in eukaryotes.

## Methods

### Bacterial strains, cell lines, and culture conditions

WT *E. coli* strain MG1655 and *hsdM* deletion K12 strain were kindly provided by Dr. Susan M Rosenberg’s lab (Baylor College of Medicine). *dam/dcm* mutant *E. coli* strain JM110 was purchased from Addgene (Bacterial strain #49763). All *E. coli* strains are cultured in typical LB broth, liquid medium at 37 °C.

### Characterization of reaction products formed from the reactions between nitrite and nucleosides in vitro

Briefly, we incubated a 90-μL reaction mixture containing 66.7 μM of individual nucleoside (dA, dC, 6mA, or 4mC), 1.0 M sodium nitrite, and 2.3% (v/v) glacier acetic acid at 37 °C. Aliquots (10 μL each) were taken out from the reaction mixtures at the indicated time points. To the aliquots were added 10 μL of 2.0 M TEAA, and the mixture was subjected immediately to HPLC analysis on a Beckman Gold HPLC system. A reversed-phase C18 column (4.6 × 250 mm, 5 μm in particle size) was used for the separation. Mobile phases A and B were 50 mM triethylammonium acetate (pH6.8) and 30% acetonitrile in mobile phase A, respectively, and the flow rate was 0.8 mL/min. The following gradients were used for the separation: 33–55.5% B in 25 min for the 2′-deoxyadenosine reaction mixture, 45–80% B in 30 min for the 6mA reaction mixture, 30–45% B in 22.5 min for the 2′-deoxycytidine reaction mixture, and 35–80% in 30 min for the 4mC reaction mixture. The LC fractions were identified using ESI-MS and MS/MS on a LCQ Deca XP mass spectrometer (Thermo) operated in positive-ion mode (Fig. S2, S4, S6, and S8).

### NT-seq for oligonucleotides

Sequences of all oligonucleotides used in this study are available at Additional file 6: Table S5. 6mA modified (156nt with one 6mA at position 60) and unmodified control oligos were synthesized at GeneLink, Inc. All other oligos, including 5mC modified oligo (100 nt with one 5mC at position 52), were synthesized at Sigma. 4mC modified oligo (91bp) was generated by treating dsDNA oligo with BamHI methyltransferase (methylates GGATCC motif at the first cytosine base) according to the manufacturer’s instructions (NEB, M0223S). BamHI methyltransferase-treated dsDNA was further treated by BamHI-HF restriction enzyme according to the manufacturer instructions to eliminate DNA with incomplete 4mC methylation. DNA oligo was first annealed to the equal number of protective oligos at both ends in 0.2 M NaCl (95 °C for 2 min, 25 °C for 5 min, and held at 4 °C with ramp rate for temperature decreasing at 0.1 °C/s). In total, 20 pmol of annealed DNA was treated with 1 M sodium nitrite (NaNO_2_) and 2.3% (v/v) acetic acid (AcOH) at 37 °C for 4–5 h. Nitrite-treated DNA was purified using Oligo Clean & Concentrator Kits (Zymo Research, D4060). Illumina TruSeq adaptor was added to oligo by PCR amplification using Taq DNA polymerase (NEB, M0490S) (cycle number was determined by qPCR with iTaq polymerase (BioRad, 1725121)). The indexed library was constructed by NEBNext Ultra™ II DNA Library Prep Kit for Illumina (NEB, E7645S). Samples were sequenced using NextSeq 500.

### NT-seq for genomic DNA

*Helicobacter pylori* JP26 genomic DNA was kindly provided by Dr. Gang Fang’s lab (Icahn School of Medicine at Mount Sinai). *E. coli* genomic DNA was isolated using DNeasy Blood and Tissue Kit according to the manufacturer’s instructions (Qiagen, 69506). Microbial community standard DNA was purchased from Zymo Research (D6306). One microgram of genomic DNA was first fragmentized to 100–300 bp using Covaris S220 Focused-ultrasonicator. Fragmented gDNA was ligated to TruSeq adaptor using NEBNext Ultra™ II DNA Library Prep Kit for Illumina (NEB, E7645S). Unmodified control DNA was made by amplifying adaptor-ligated DNA using Q5 DNA polymerase (NEB, M0492S). Both native and amplified DNA was first annealed to excessive protective adaptor sequences in 5 mM adaptor oligos (F-RC: AGATCGGAAGAGCGTCGTGTAGGGAAAGAGTGT, R: CTGGAGTTCAGACGTGTGCTCTTCCGATCT) and 0.2 M NaCl (95 °C for 2 min and hold at 37 °C with ramp rate for temperature decreasing at 1 °C/s) and then treated by 1 M NaNO_2_ and 2.3% (v/v) AcOH at 37 °C for 4 h. Nitrite-treated DNA was purified using Oligo Clean & Concentrator Kits (Zymo Research, D4060). The indexed library was constructed using Taq DNA polymerase (NEB, M0490S) (cycle number was determined by qPCR with (BioRad, 1725121)). Samples were sequenced using NextSeq 500.

### 6mA DIP-NT-seq

One microgram adaptor-ligated *E. coli* genomic DNA was denatured at 95 °C for 10 min and ice bath immediately for 10 min. Single-stranded DNA fragments were immunoprecipitated with 6mA antibodies (CST, D9D9W) overnight at 4 °C. Methylated DNA capture, wash, and elution were performed using the hMeDIP kit according to the manufacturer’s instructions (Active motif, 55010). Eluted DNA was treated by 2 μl 1 mg/ml proteinase K at 50 °C for 30 min and purified by Oligo Clean & Concentrator Kits (Zymo Research, D4060) to remove the remaining antibodies. A small proportion of input and enriched DNA was used to construct traditional 6mA DIP-seq libraries. The remaining enriched DNA was used to perform NT-seq as described above.

### NT-seq data analysis

#### Preprocess

Analysis workflow is shown in Additional file 1: Fig. S11. The scripts used for analysis are available at GitHub (https://github.com/TaoLabBCM/NT-seq) [42] and Zenodo (https://zenodo.org/record/6540299#.Ynwdt5PML_s) [43]. Briefly, sequencing reads were trimmed using Cutadapt (v1.18) [44] to remove adaptors. Duplicated reads were removed using BBMAP (v38.84) [45], Clumpify package (This step is omitted for oligo data analysis). To align all nitrite treatment converted reads (A to G and C to T mutations) to reference genome, FASTQ reads and FASTA reference were converted to AT-only format (convert all purine (A/G) to A and all pyrimidine (C/T) to T). Converted FASTQ reads were aligned to converted reference using Bowtie2 (v2.3.5.1) [46]. AT-only SAM files were converted back to SAM files with original reads using custom python scripts. NM (number of mismatches between the sequence and reference) and MD (string encoding mismatched reference bases) tags in SAM files were recalculated using Samtools (1.9) [47] calmd command to obtain mutation pattern of original reads. The alignments in SAM files were further filtered by recalculated NM and MD tags to remove unconverted reads and reads with unwanted base mutation (number of mismatch ≥ 2, number of mismatches at A or C position/total number of mismatches ≥ 0.8). Base count at each genomic location was generated by Igvtools (v2.5.3) [48] count command using filtered SAM files, and the base count files were used to calculate A to G ratio and C to T ratio of native and amplified samples at each position using custom python scripts.

#### Methylation motif identification

For all possible methylation motif combinations (4mer, 5mer, and 6mer motifs for 6mA, 5mC, and 4mC; bipartite type I RM system motifs for 6mA), an average A to G or C to T ratio were calculated. A to G ratio and C to T ratio data were first normalized by dividing average A to G and C to T ratio between native and amplified samples and then log-transformed. Motifs were filtered by requiring at least 50 motif loci to be covered with at least 200 reads. Motifs passing filters were then used to generate the scatter plots for motif identification.

### 6mA DIP-seq analysis

6mA DIP-seq datasets for *L. fermentum* and *E. coli* were downloaded from NCBI SRA with Bioproject number PRJNA477598. Raw reads were first trimmed using Cutadapt (1.18) [44] to remove adaptors. Trimmed reads were aligned to the reference genome (Additional file 7: Table S6) using bowtie2 (v2.3.5.1) [46]. 6mA peaks were identified using MACS3 (3.0.0a6) with default parameters for Oxytricha DIP-seq and parameters: *--extsize 50 --keep-dup all* for *L. fermentum* DIP-seq. Motif enrichment analysis of 6mA peaks in *L. fermentum* was performed using DREME in MEME suite (v5.0.5, shuffled 6mA peaks as negative control) [49].

### Bisulfite sequencing analysis

Bisulfite sequencing data for bacterial species in Zymomics microbial community reference were downloaded from NCBI SRA with Bioproject number PRJNA477598. Raw reads were first trimmed using Cutadapt (v1.18) [44] to remove adaptors. Downstream alignment, deduplication, and methylation extraction for each cytosine position were performed using Bismark (v0.23.1) [50]. Cytosine methylation motifs were identified using custom python scripts.

### Comparing DIP-NT-seq to SMRT-seq

SMRT-seq 6mA and 4mC results for *E. coli* strain MG1655 were obtained from the published dataset MethSMRT (http://sysbio.gzzoc.com/methsmrt) [35]. SMRT-seq 6mA results were obtained from GEO: GSE94421. SMRT-seq data for *H. pylori* JP26 were obtained from SRA: SRP109061 (SRR5678374) and analyzed using SMRT Link (v10.1) with default parameters. Comparison between SMRT-seq and DIP-NT-seq in *E. coli* MG1655 was performed by overlapping 6mA sites detected by SMRT-seq or DIP-NT-seq with Dam and M. EcoKI 6mA motif sites. Only genomic locations passing sequencing depth filter (25X) in DIP-NT-seq were considered.

## Supplementary Information

**Additional file 1: Fig. S1-S9.** Characterization of reaction products formed from the reactions between nitrite and nucleosides in vitro. **Fig. S10.** DNA degradation comparison between nitrite treatment and bisulfite treatment. **Fig. S11.** Analysis workflow for NT-seq. **Fig. S12-16.** Additional information for methylation detection in oligonucleotides, *E. coli* MG1655 genome, *H. pylori* JP26 genome, and microbial community reference by NT-seq. **Fig. S17.** NT-seq performance in detecting 6mA, 4mC, and 5mC at single-base resolution in *H. pylori* JP26 genome. **Fig. S18.** Additional information for single-base detection of 6mA by DIP-NT-seq in *E. coli*. **Fig. S19.** Performance comparison between DIP-NT-seq and DIP-seq/6mACE-seq for 6mA detection in *E. coli* genome. **Fig. S20.** Comparison between SMRT-seq detected 6mA motifs and Nanopore sequencing detected 6mA motifs by nanodisco in a mouse gut microbiome sample.

**Additional file 2: Table S1.** Comparison between protected and unprotected oligo under the same nitrite treatment.

**Additional file 3: Table S2.** Nitrite treatment condition optimization by qPCR.

**Additional file 4: Table S3.** 5mC motif detected from bisulfite sequencing in eight bacterial species in microbial community standard.

**Additional file 5: Table S4.** PCR duplication rate of unenriched and 6mA DIP enriched library.

**Additional file 6: Table S5.** Oligos and primers used in this study.

**Additional file 7: Table S6.** Reference genome used in this study.

**Additional file 8.** Review history.

## Data Availability

All the NT-seq data that were generated in this study are stored in Gene Expression Omnibus database under NCBI with the accession number GSE184374 [51]. DIP-seq data and bisulfite sequencing datasets are downloaded from NCBI SRA with Bioproject number PRJNA477598 [52]. SMRT-seq for *Oxytricha* and *H. pylori* JP26 datasets are obtained from GEO: GSE94421 [53] and SRA: SRP109061 (SRR5678374) [54]. The scripts used for NT-seq analysis are available under MIT license at GitHub (https://github.com/TaoLabBCM/NT-seq) [42] and Zenodo (https://zenodo.org/record/6540299#.Ynwdt5PML_s) [43].
