# Supplementary material for: NT-seq: a chemical-based sequencing method for genomic methylome profiling
Source: Genome Biol. 2022 May 30;23:122. doi: 10.1186/s13059-022-02689-9 (PMC9150344; doi:10.1186/s13059-022-02689-9)
Supplement: Supplementary file 1 — Additional file 1: Fig. S1-S9. Characterization of reaction products formed from the reactions between nitrite and nucleosides in vitro. Fig. S10. DNA degradation comparison between nitrite treatment and bisulfite treatment. Fig. S11. Analysis workflow for NT-seq. Fig. S12-16. Additional information for methylation detection in oligonucleotides, E. coli MG1655 genome, H. pylori JP26 genome, and microbial community reference by NT-seq. Fig. S17. NT-seq performance in detecting 6mA, 4mC, and 5mC at single-base resolution in H. pylori JP26 genome. Fig. S18. Additional information for single-base detection of 6mA by DIP-NT-seq in E. coli. Fig. S19. Performance comparison between DIP-NT-seq and DIP-seq/6mACE-seq for 6mA detection in E. coli genome. Fig. S20. Comparison between SMRT-seq detected 6mA motifs and Nanopore sequencing detected 6mA motifs by nanodisco in a mouse gut microbiome sample. [file 13059_2022_2689_MOESM1_ESM.docx]

# Supplementary Figures


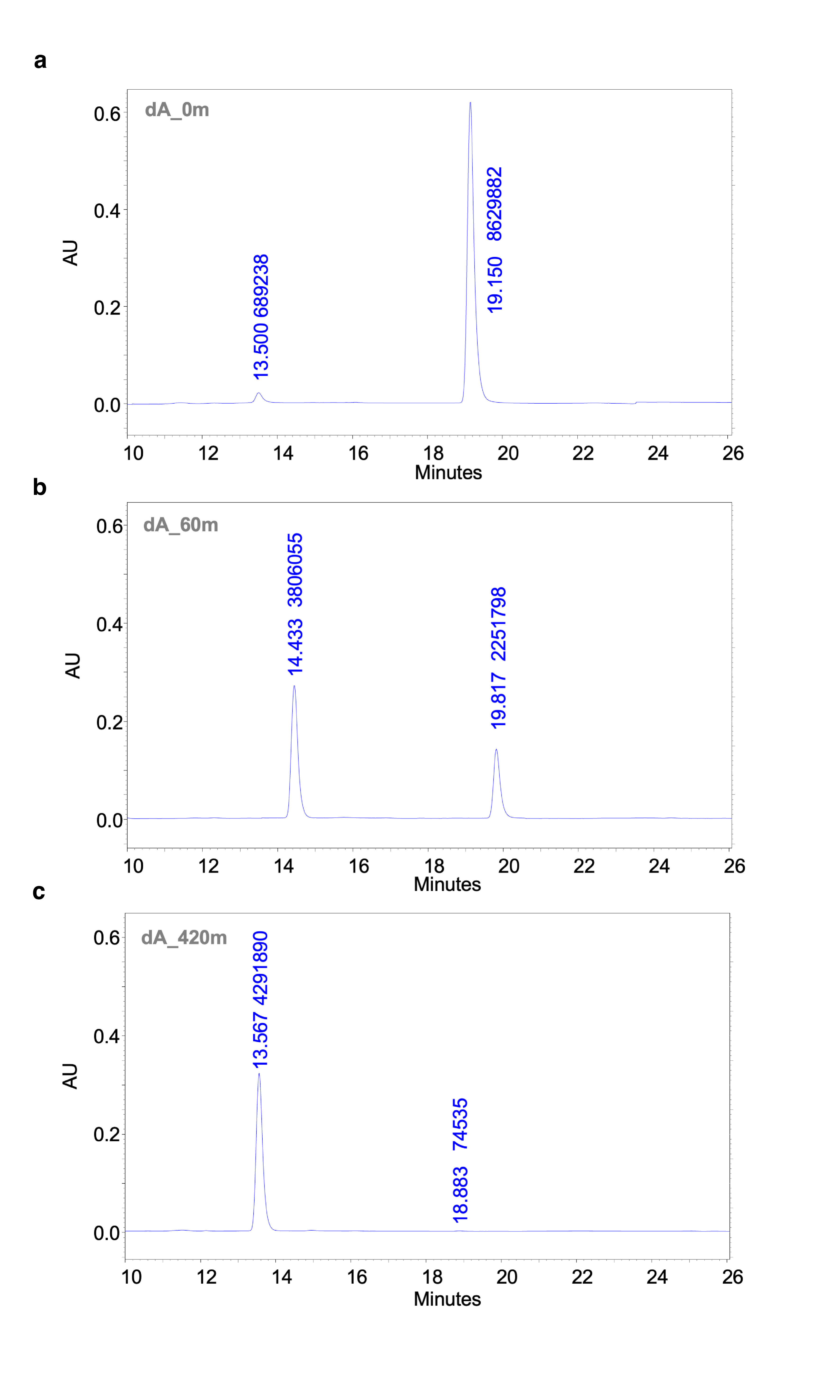


Fig. S1**. HPLC traces for the separations of the reaction mixture of 2′-deoxyadenosine (dA) with nitrite at different time points.** **a**, immediately after nitrite addition (0 min). **b**, 60 min after nitrite addition **c**, 7 hrs after nitrite addition. Labeled above the peaks are the retention times and peak areas.


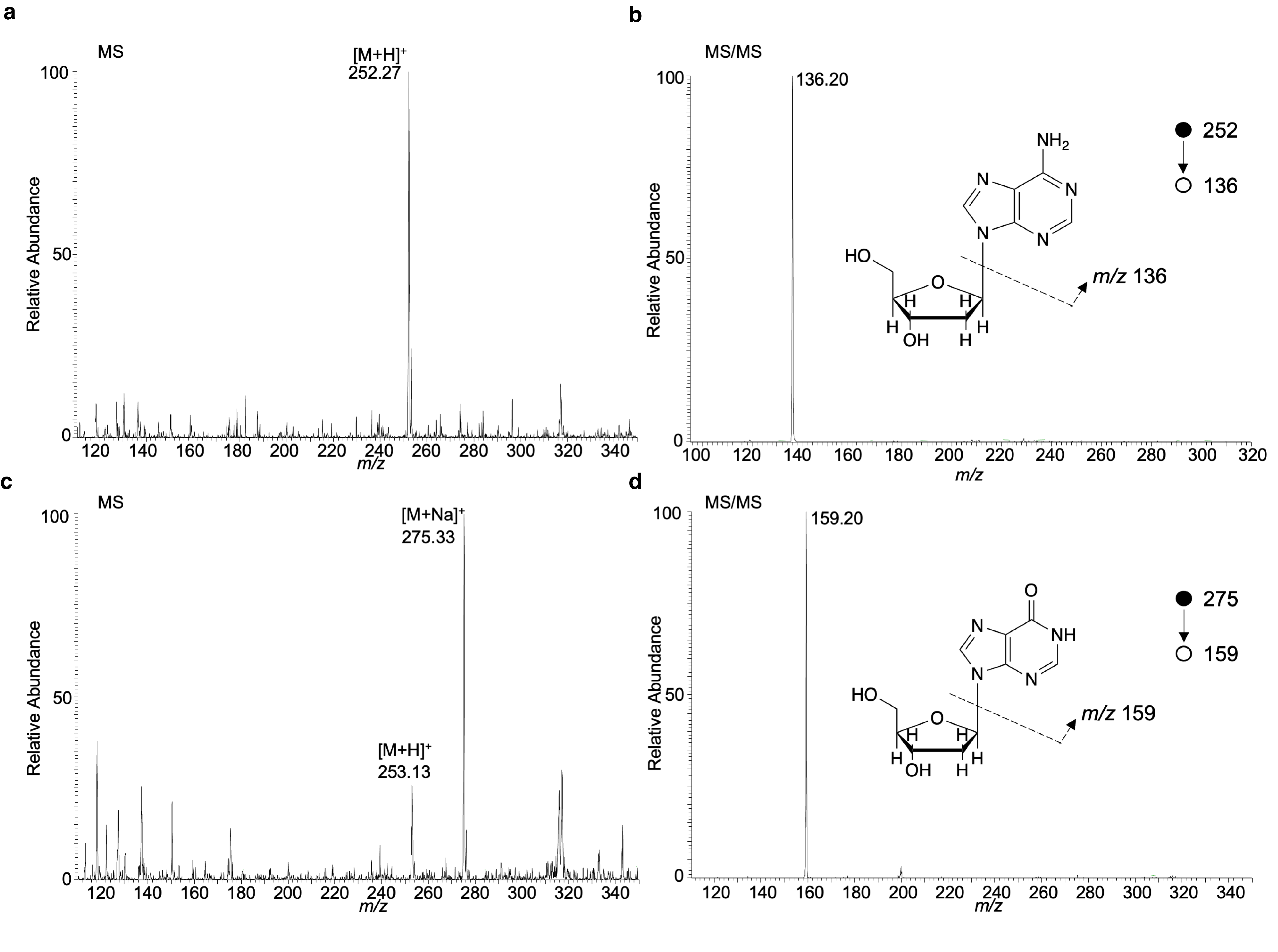


Fig. S2**. Mass spectrometric characterizations of dA and its deamination product.** **a**, Positive-ion ESI-MS of the 19.8-min fraction (unreacted dA) in Fig. S1a. **b**, Product-ion spectrum (MS/MS) for the [M+H]^+^ ion (*m/z* 252) of dA (the 19.8-min fraction in Fig. S1a). **c**, Positive-ion ESI-MS of the 13.6-min fraction (the deamination product of dA, i.e., 2′-deoxyinosine) in Fig. S1c. **d**, MS/MS for the [M+Na]^+^ ion (*m/z* 275) of 2′-deoxyinosine (the 13.6-min fraction in Fig. S1c).


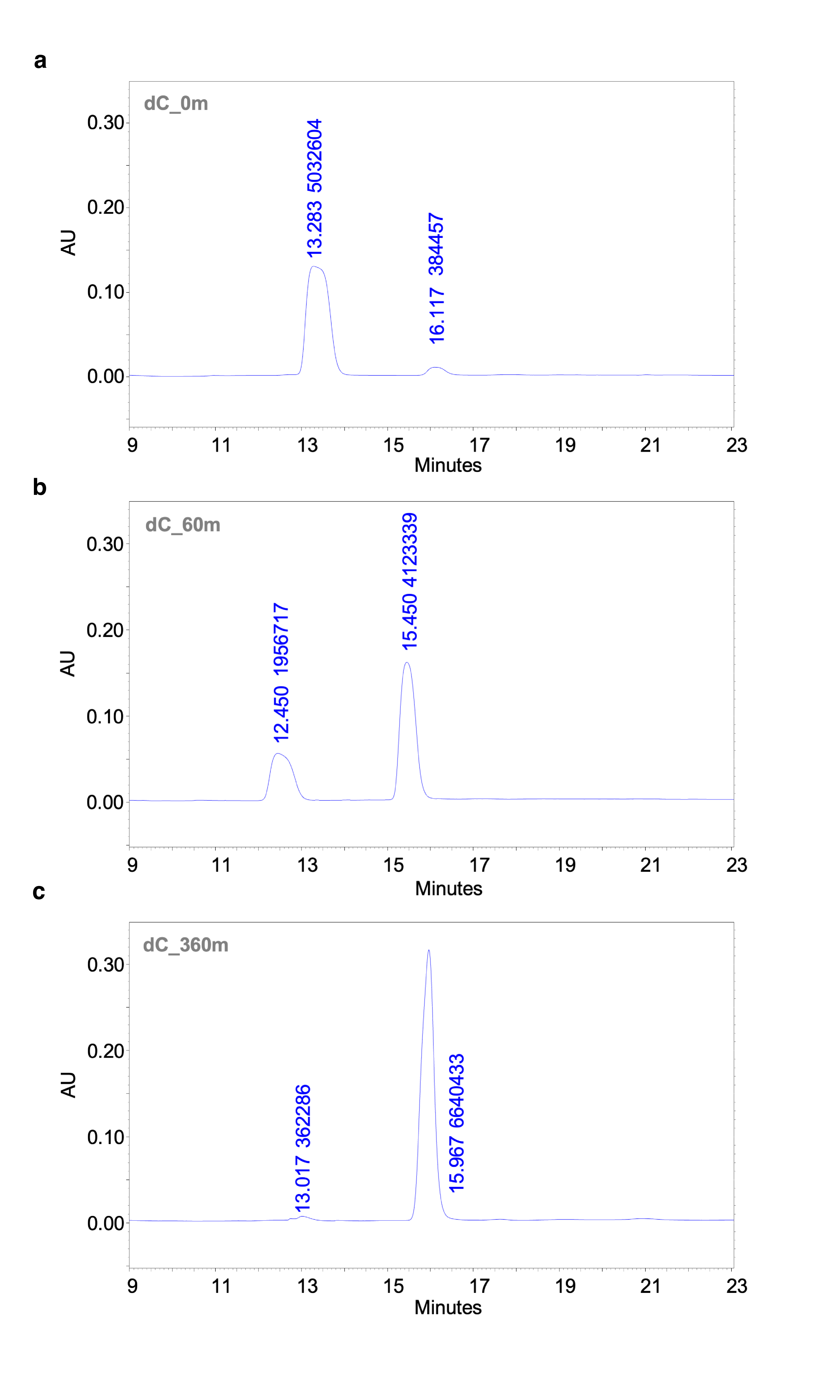


Fig. S3**. HPLC traces for the separations of reaction mixture of 2′-deoxycytidine (dC) with nitrite at different time points.** **a**, immediately after nitrite addition (0 min). **b**, 60 min after nitrite addition **c**, 6 hrs after nitrite addition. Labeled above the peaks are the retention times and peak areas.


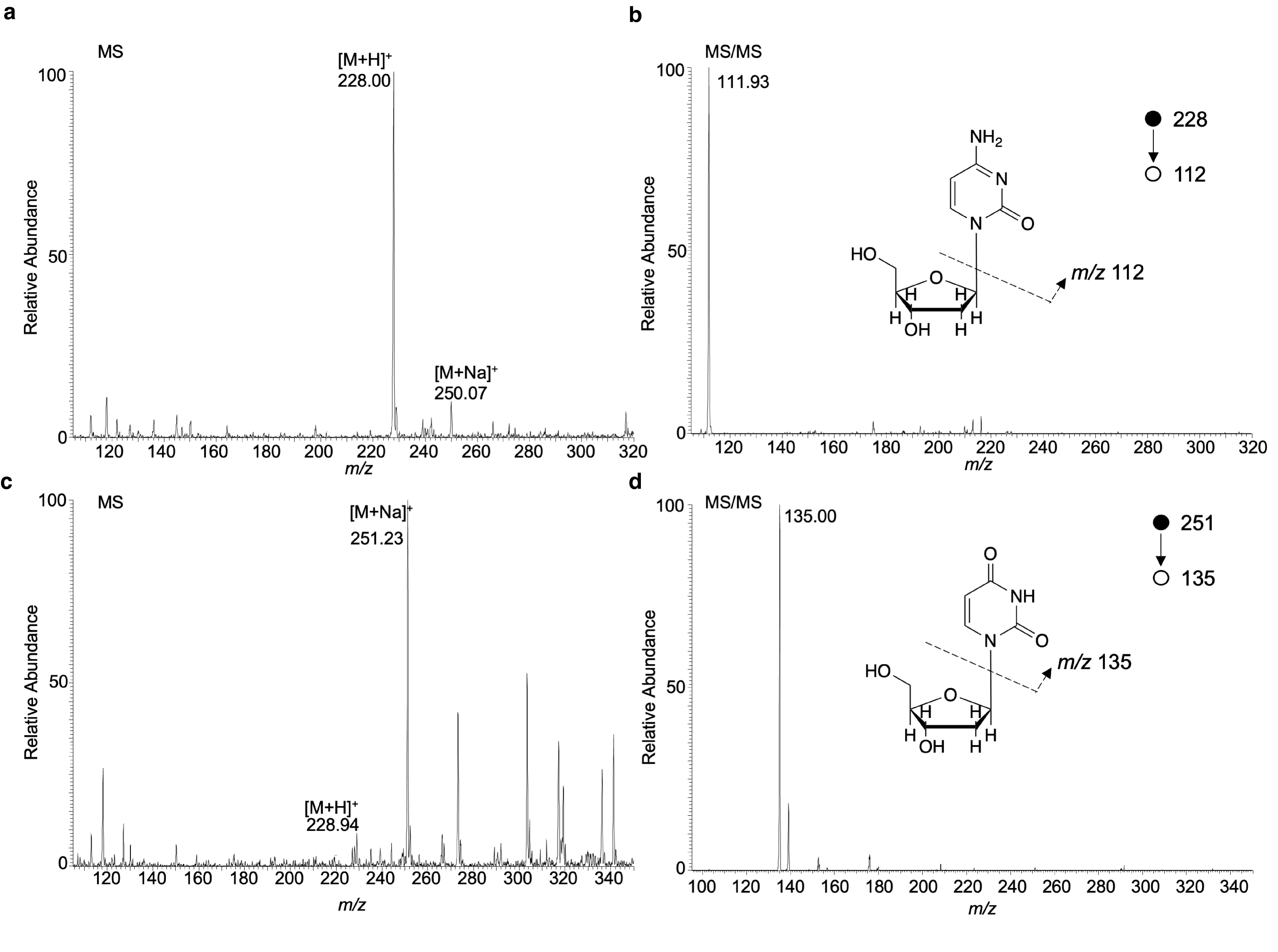


Fig. S4**. Mass spectrometric characterizations of dC and its deamination product.** **a**, Positive-ion ESI-MS of the 13.3-min fraction (unreacted dC) in Fig. S5a. **b**, MS/MS for the [M+H]^+^ ion (*m/z* 228) of dC (the 13.3-min fraction in Fig. S5a). **c**, Positive-ion ESI-MS of the 16-min fraction (the deamination product of dC, i.e., 2′-deoxyuridine) in Fig. S5c. **d**, MS/MS for the [M+Na]^+^ ion (*m/z* 251) of 2′-deoxyuridine (the 16-min fraction in Fig. S5c).


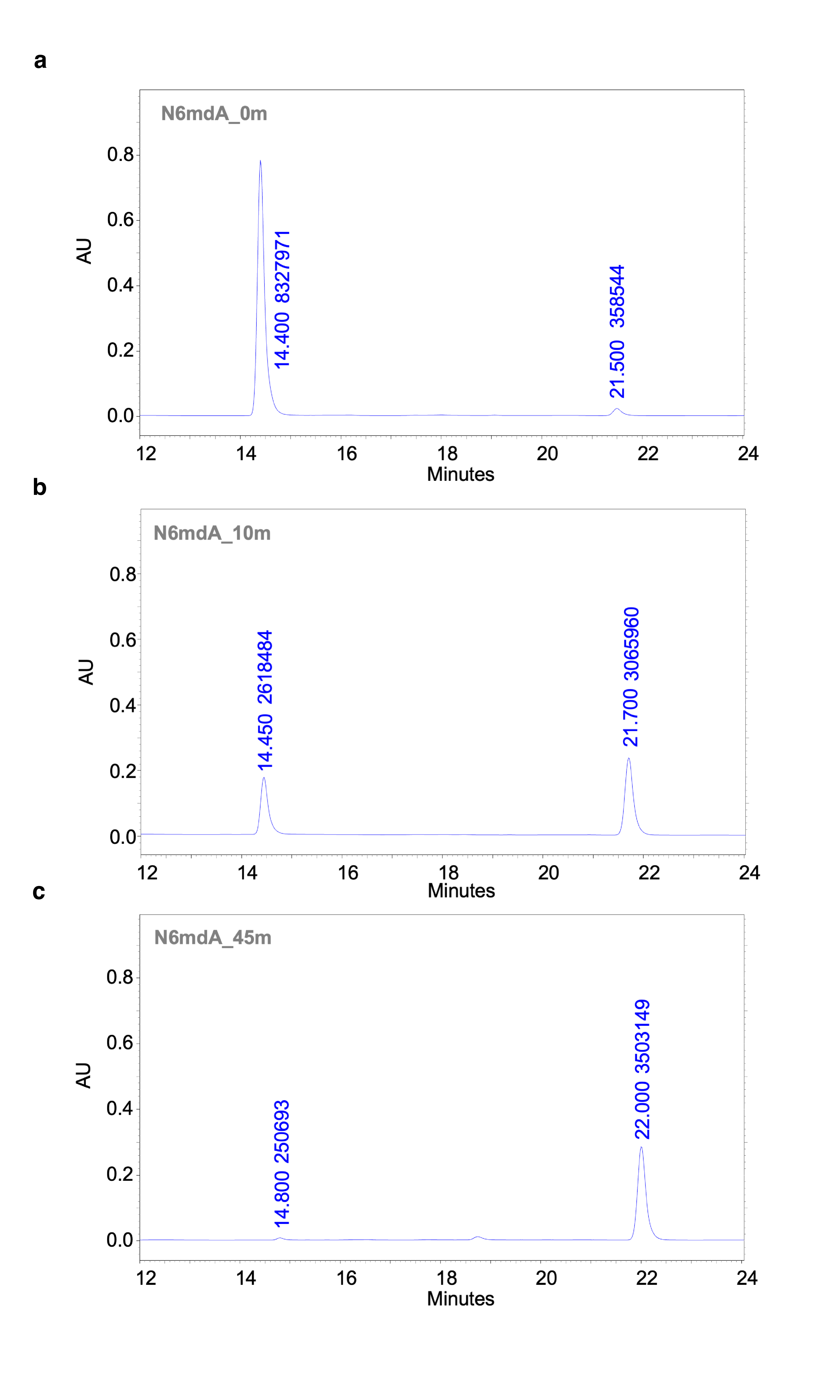


Fig. S5**. HPLC traces for the separations of reaction mixture of 6mdA with nitrite at different time points.** **a**, immediately after nitrite addition (0 min). **b**, 10 min after nitrite addition **c**, 45 min after nitrite addition. Labeled above the peaks are the retention times and peak areas.


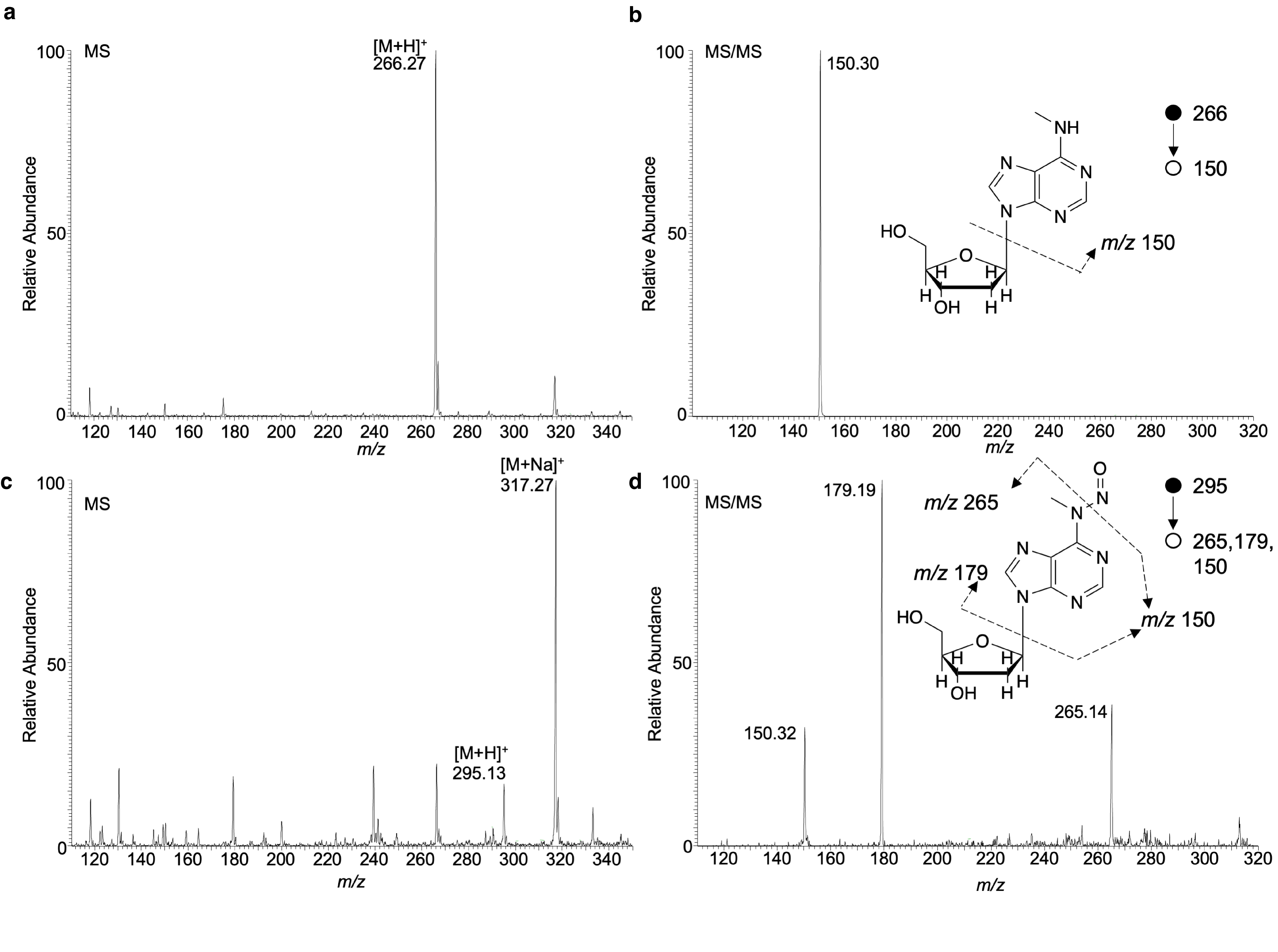


Fig. S6**. Mass spectrometric characterizations of 6mdA and its N-nitrosylated 6mdA.** **a**, Positive-ion ESI-MS of the 14.4-min fraction (unreacted 6mdA) in Fig. S3a. **b**, MS/MS for the [M+H]^+^ ion (*m/z* 266) of 6mdA (the 14.4-min fraction in Fig. S3a). **c**, Positive-ion ESI-MS of the 22-min fraction (6mdA-NO) in Fig. S3c. **d**, MS/MS for the [M+H]^+^ ion (*m/z* 295) of 6mdA-NO (the 22-min fraction in Fig. S3c).


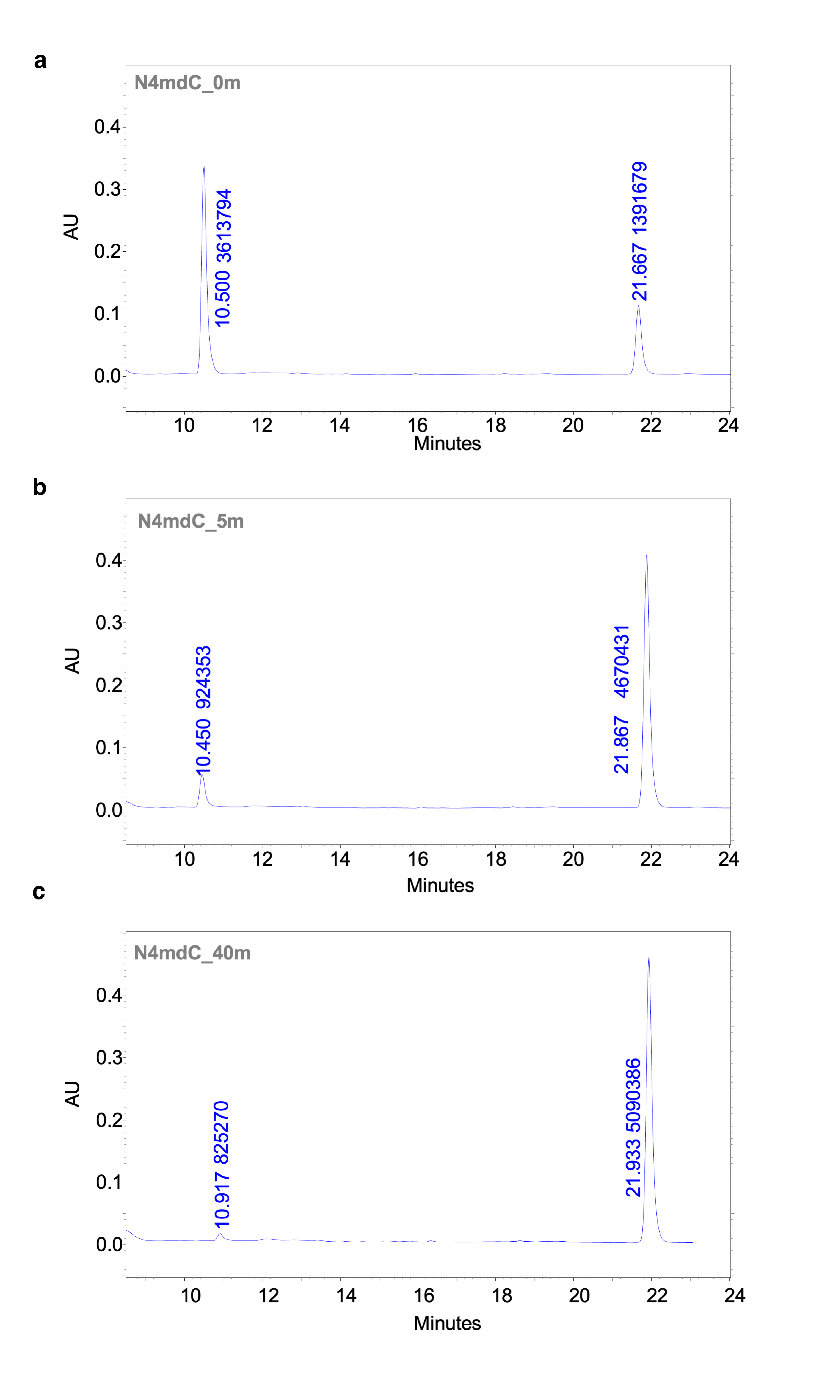


Fig. S7**. HPLC traces for the separations of reaction mixture of 4mdC with nitrite at different time points.** **a**, immediately after nitrite addition (0 min). **b**, 5 min after nitrite addition **c**, 40 min after nitrite addition. Labeled above the peaks are the retention times and peak areas.


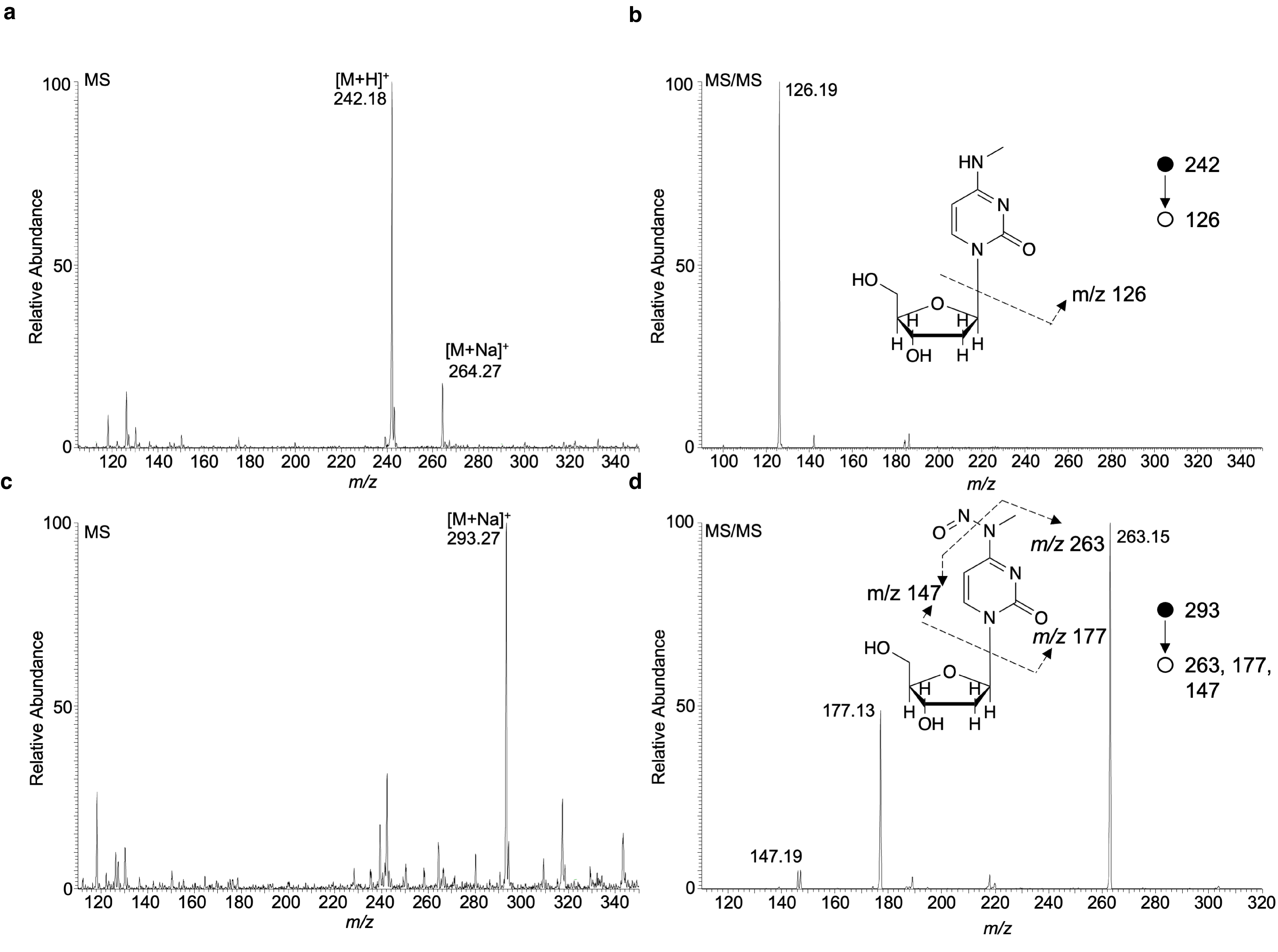


Fig. S8**. Mass spectrometric characterizations of 4mdC and N-nitrosylated 4mdC.** **a**, Positive-ion ESI-MS of the 10.5-min fraction (unreacted 4mdC) in Fig. S7a. **b**, MS/MS for the [M+H]^+^ ion (*m/z* 242) of 4mdC (the 10.5-min fraction in Fig. S7a). **c**, Positive-ion ESI-MS of the 21.9-min fraction (4mdC-NO) in Fig. S7c. **d**, MS/MS for the [M+Na]^+^ ion (*m/z* 293) of 4mdC-NO (the 21.9-min fraction in Fig. S7c).


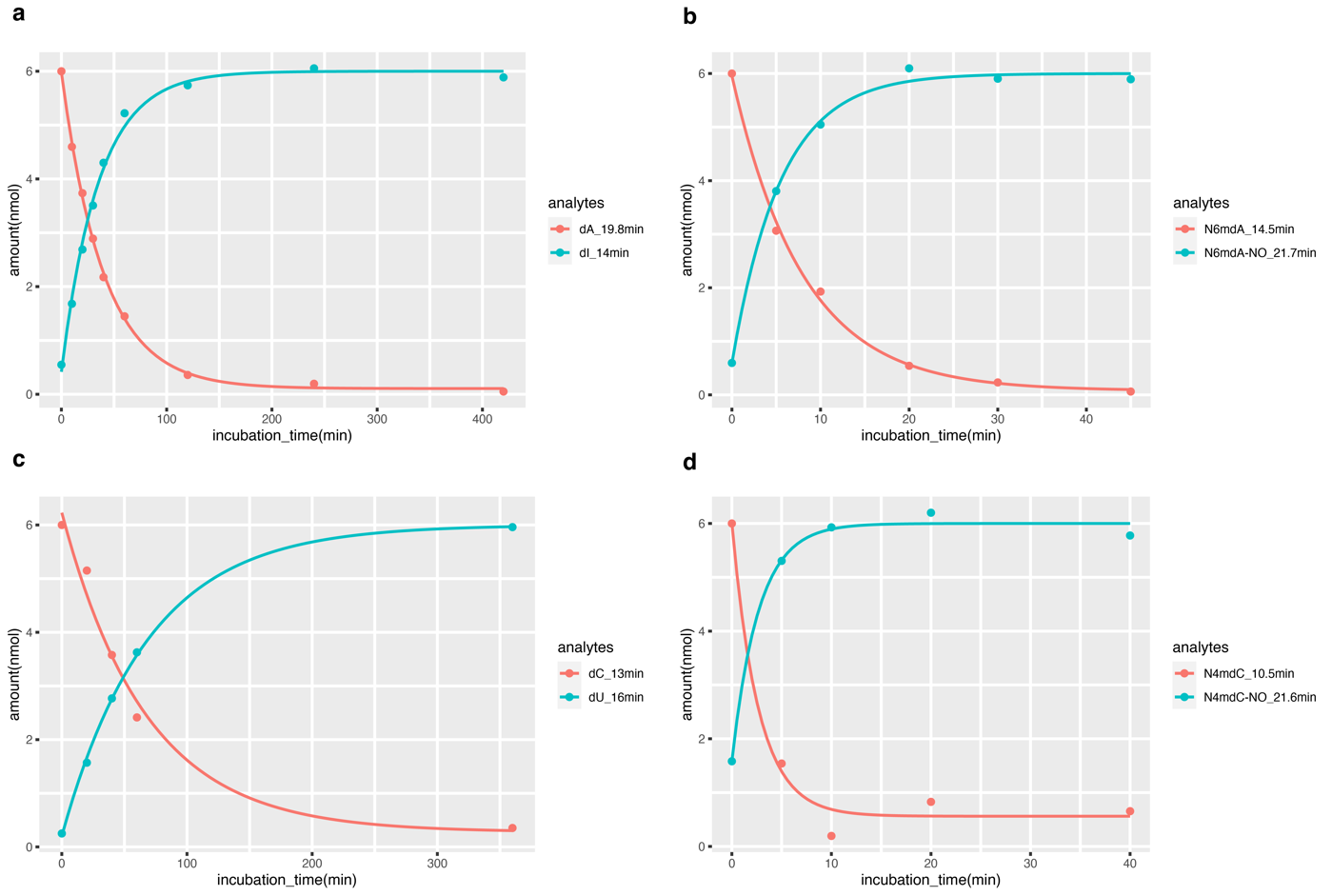


Fig. S9**. Dynamics of dA, 6mdA, dC, 4mdC and the dominant reaction products formed from nitrite treatment.** **a-d**, plots showing the dynamic changes in the amounts of: **a**, 2′-deoxyadenosine (dA) and 2′-deoxyinosine (dI); **b**, 6mdA and 6mdA-NO; **c**, 2′-deoxycytidine (dC) and 2′-deoxyuridine (dU); **d**, 4mdC and 4mdC-NO, at different time points following nitrite addition.


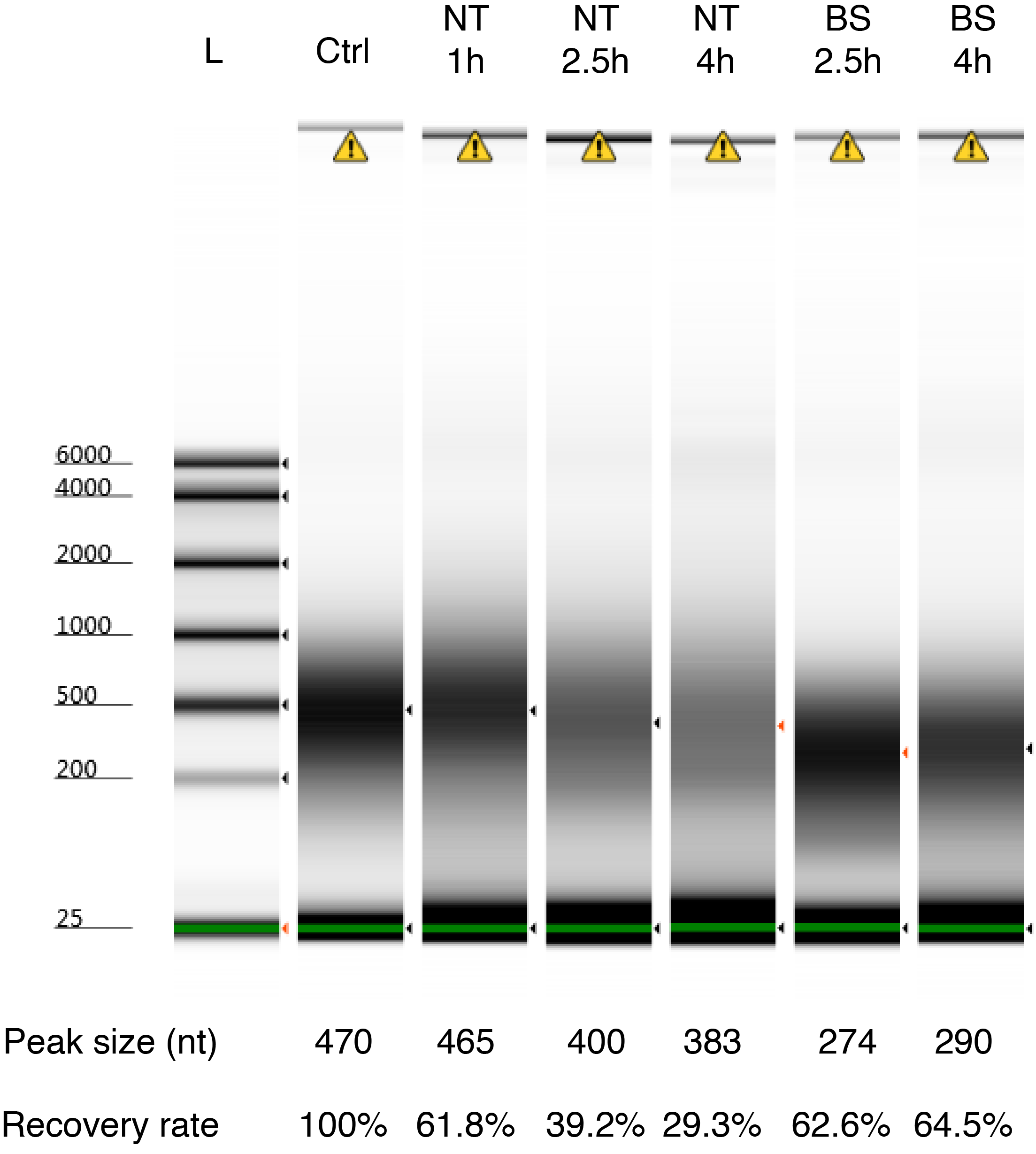


Fig. S10**. DNA degradation comparison between nitrite treatment and bisulfite treatment.** Tapestation RNA ScreenTape analysis of untreated (ctrl), nitrite treated (NT), and bisulfite treated (BS) 1μg ssDNA using sonicated (~500bp, ctrl) genomic DNA from 293T cells. ssDNA length distribution and recovery rate indicate that nitrite treatment causes less fragmentation but more DNA degradation than bisulfite treatment. Nitrite treatment: 1M NaNO_2_, 2.3% AcOH and incubate at 37 °C for 1, 2.5, or 4 hours; Bisulfite treatment was performed using EZ DNA Methylation-Gold kit (D5005) from Zymo Research (incubate at 64 °C for 2.5 or 4 hours).

**
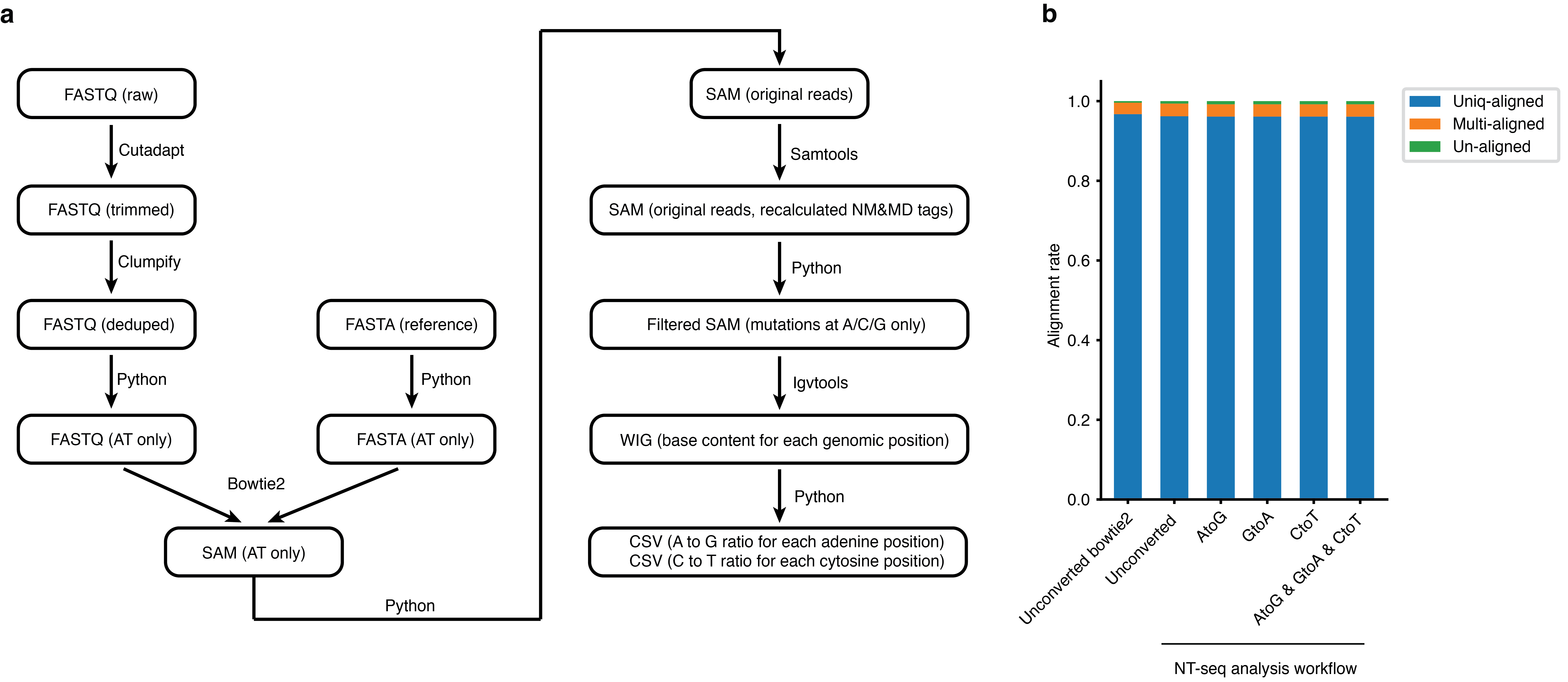
**

Fig. S11**. Analysis workflow for NT-seq. a**, PCR duplicates were removed to accurately calculate A to G or C to T ratio. To align nitrite converted DNA to the original reference genome, we converted the FASTQ reads and the FASTA reference to AT only format (A represents purine (A/G) and T represents pyrimidine (C/T)). AT only SAM files were converted back to original reads and filtered to make sure only reads with A to G or C to T mutations were preserved. Filtered SAM files were used to generate base content at each genomic position, which was used to calculate A to G and C to T ratio. **b**, Alignment results of *E. coli* MG1655 strand-specific WGS data (unconverted or artificially introduced AtoG/GtoA/CtoT base change for all FASTQ reads­­ (85bp)) indicate that NT-seq analysis workflow can tolerate all possible base conversions by nitrite treatment.

**
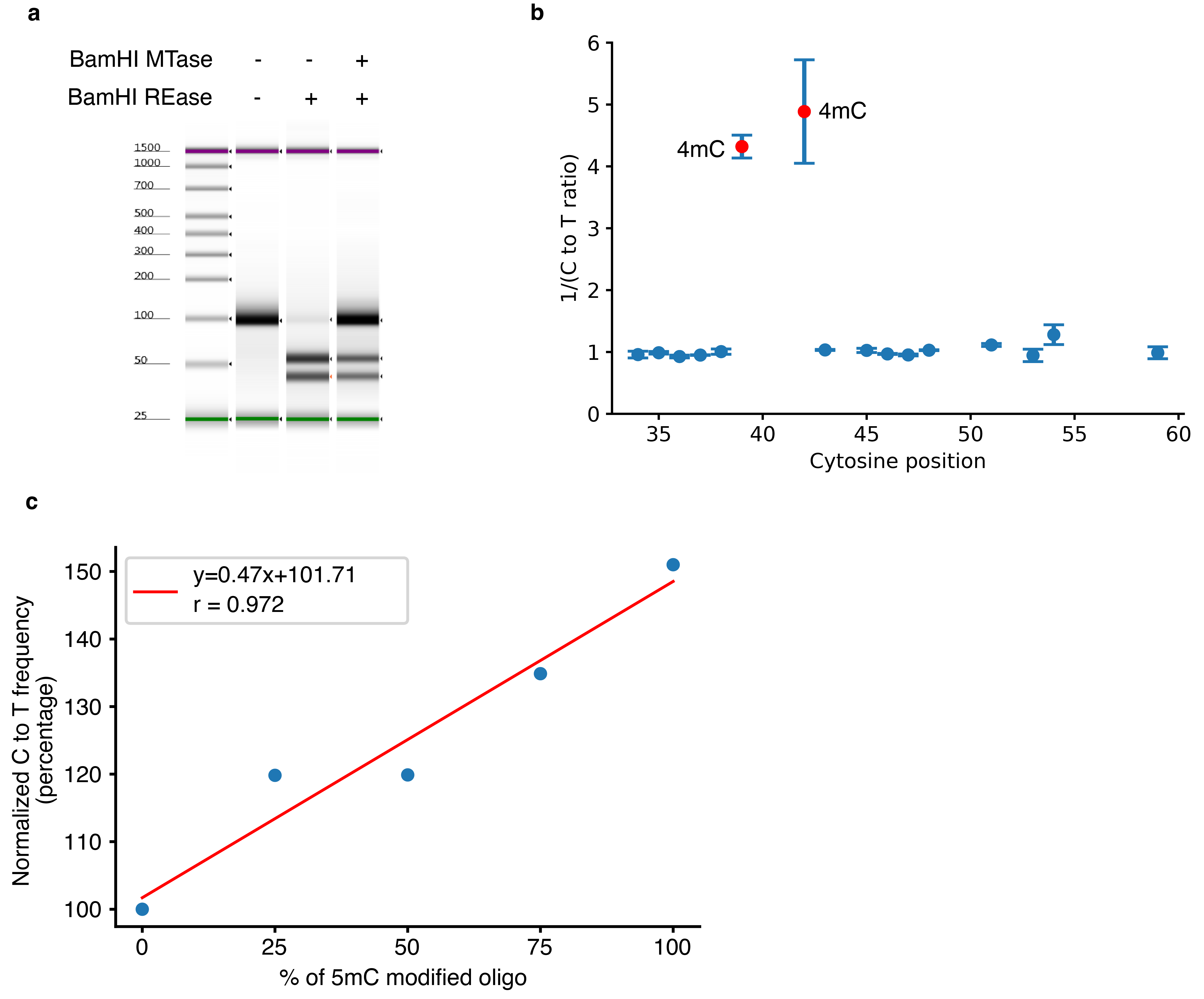
**

**﻿**Fig. S12**. Additional information for methylation detection in oligonucleotides by NT-seq. a**, Tapestation D1000 tape result indicates successful 4mC methylation by BamHI methyltransferase in oligo. **b**, The inverse of C to T ratio at cytosine sites between unmodified oligo and oligo modified by BamHI methyltransferase from another batch of the experiment. Both samples were treated and sequenced as triplicates. Dots represent the mean and error bars represent standard deviation. **c**, Correlation between normalized C to T frequency of 5mC site and the percentage of 5mC modified oligo.


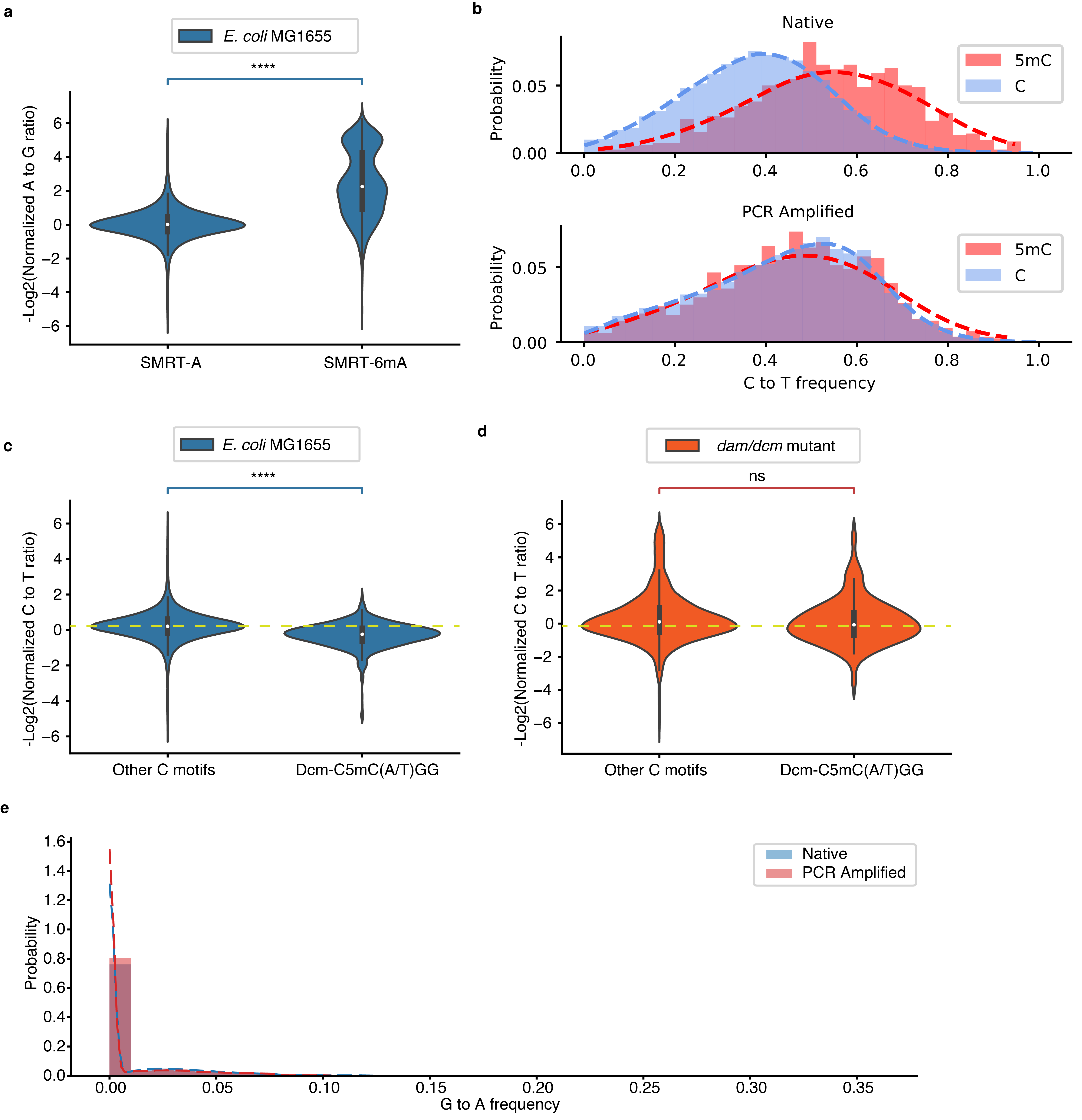


Fig. S13**. Additional information for methylation detection in *E. coli* genome by NT-seq. a**, Negative log normalized A to G ratio of 6mA sites identified by SMRT-seq in *E. coli* MG1655. **b,** C to T frequency at known 5mC sites (C5mCWGG) and unmethylated C sites in *E. coli* MG1655 genome from native and PCR amplified DNA. **c-d**, Negative log normalized C to T ratio of different 5mC motifs in *E. coli* strain MG1655 (**d**) and *dam/dcm/hsdR* mutated strain (**d**). **e**, G to A frequency at G sites in *E. coli* MG1655 genome from native and PCR amplified DNA. Only motifs with sequencing depth larger than 25X were considered for violin plots. Statistic test were performed by two-sided Mann-Whitney-Wilcoxon (MWW) test with Bonferroni correction (ns: *P* > 1.0e-3; ****: *P* <= 1.0e-6).


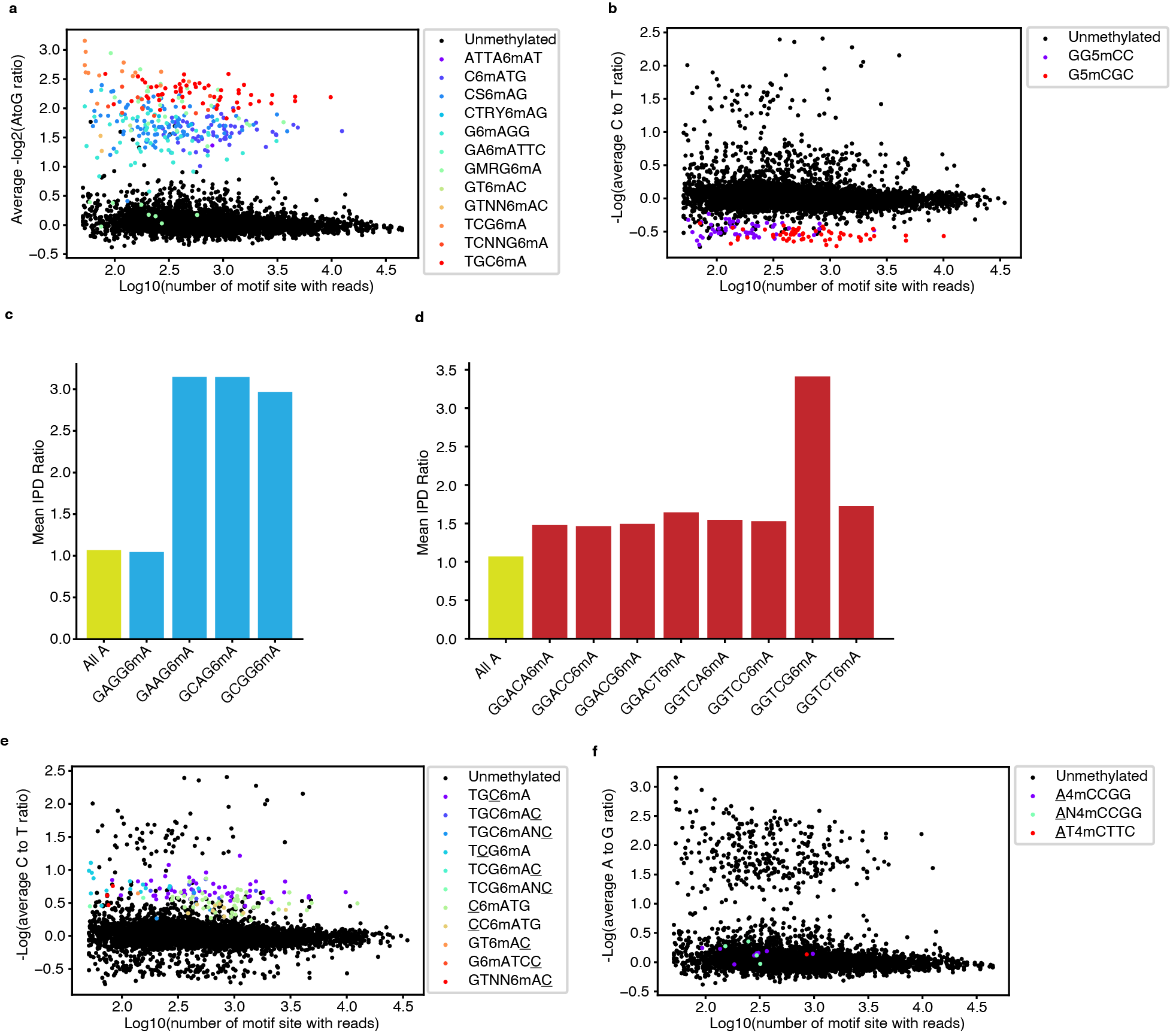


Fig. S14**. Additional information for De novo discovery of methylation motifs in *H. pylori* genome by NT-seq. a**, Scatter plot of the median difference of -Log2FC between any 4mer-6mer A motif and the remaining A sites (Previously reported 6mA motifs are labeled in color). **b**, Scatter plot of the median difference of -Log2FC between any 4mer-6mer C motif and the remaining C sites (reported 5mC motifs are labeled in color). **c**, Mean IPD Ratio of four sub-motifs of GMRG6mA from SMRT-seq. **d**, Mean IPD ratio of eight sub-motifs of GGWCN6mA from SMRT-seq. **e**, Scatter plot indicates nitrite treated 6mA can impact surrounding nucleotide incorporation. **f**, Scatter plot indicates nitrite treated 4mC does not impact surrounding nucleotide incorporation.


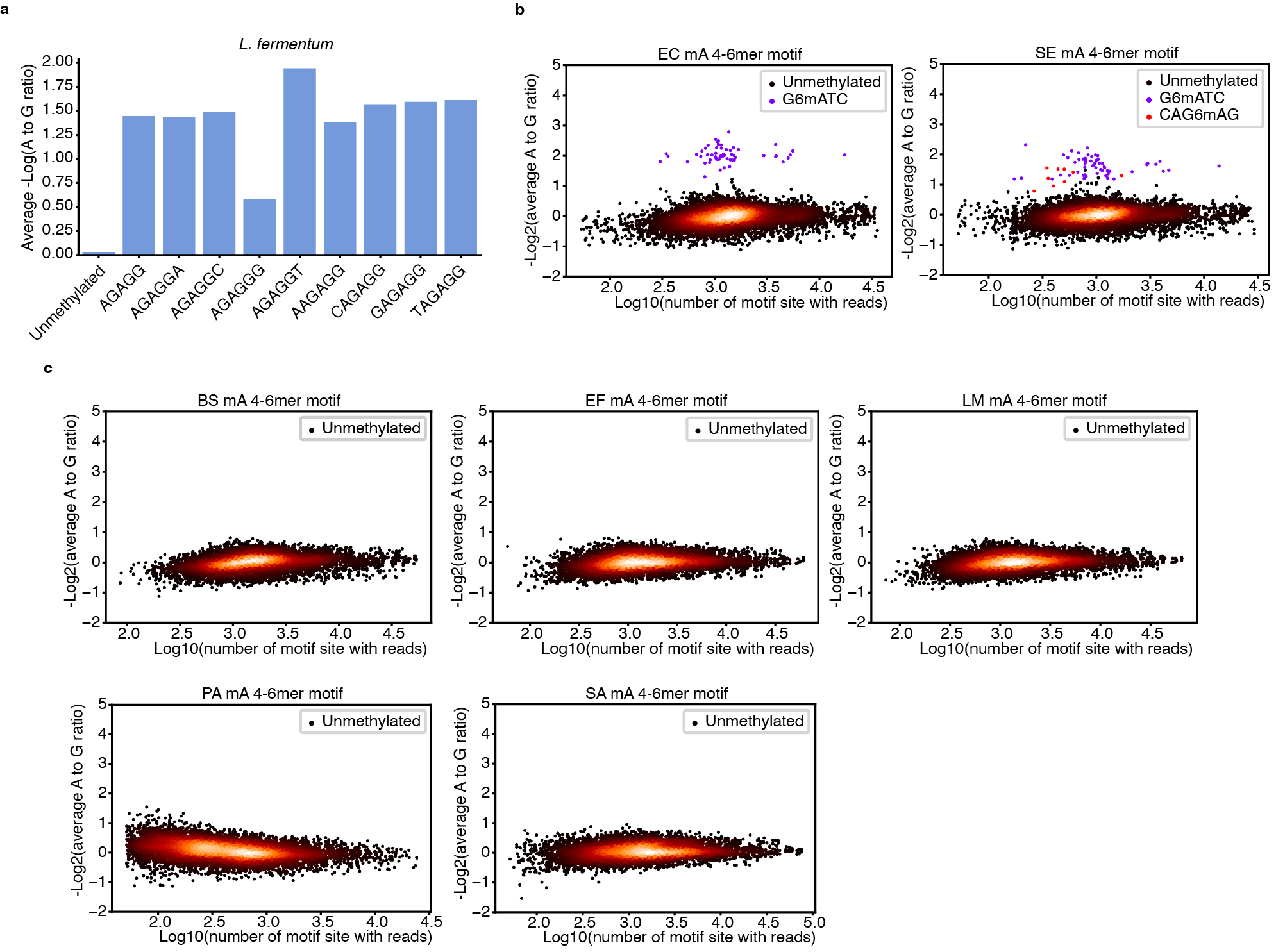


Fig. S15**. Additional information for NT-seq in microbial community reference.** **a**, Average A to G ratio at all 4-6mer sub-motifs of AG6mAGG in *L. fermentum*. **b**, Average A to G ratio of all possible 4-6mer adenine motifs in *E. coli* and *S. enterica.* **c**, Average A to G ratio of all possible 4-6mer adenine motifs in *B. subtili*, *E. faecalis*, *L. monocytogenes*, *S. aureus*, and *P. aeruginosa.*


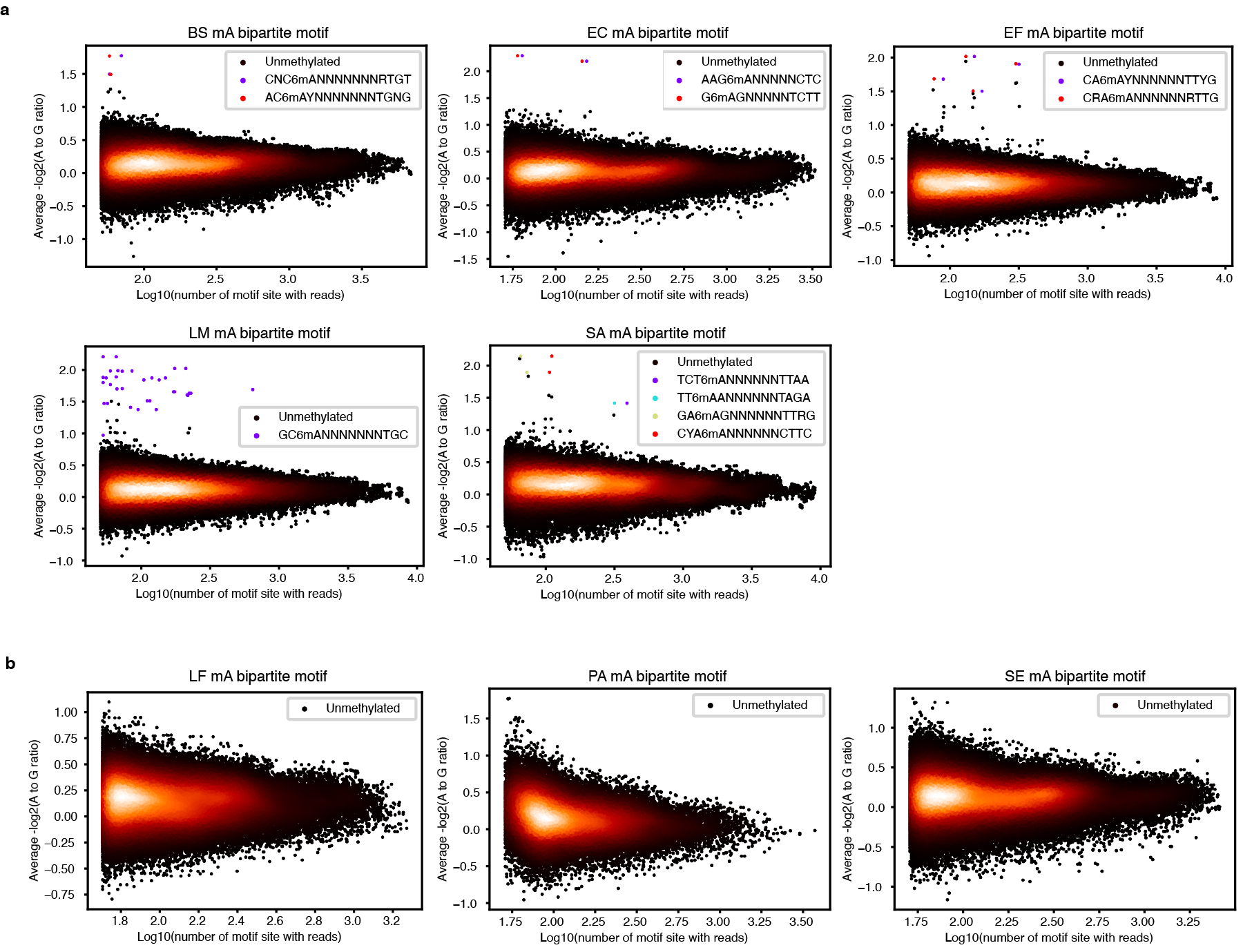


Fig. S16**. Additional information for NT-seq in microbial community reference.** **a**, Average A to G ratio of common type I RM motifs in *B. subtili*, *E. coli, E. faecalis*, *L. monocytogenes*, and *S. aureus.* **b**, Average A to G ratio of common type I RM motifs in *L. fermentum*, *P. aeruginosa*, and *S. enterica.*


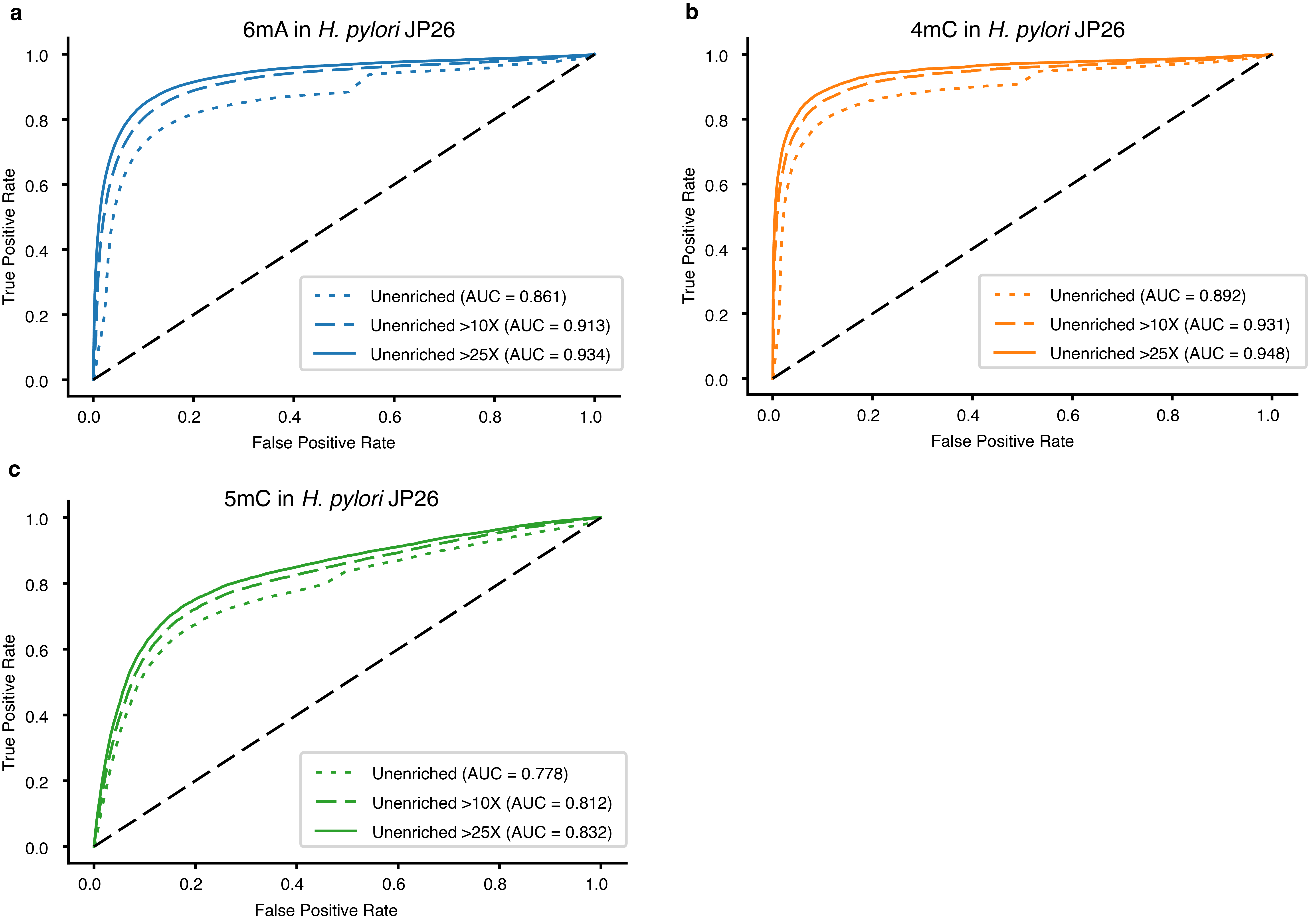


Fig. S17**. NT-seq performance in detecting 6mA, 4mC, and 5mC at single-base resolution in *H. pylori* JP26 genome**. **a**, ROC curves show the performance of NT-seq in detecting 6mA at single-base resolution using different sequencing depth filtering. **b**, ROC curves show the performance of NT-seq in detecting 4mC at single-base resolution using different sequencing depth filtering. **c**, ROC curves show the performance of NT-seq in detecting 5mC at single-base resolution using different sequencing depth filtering. Methylation sites within NT-seq and SMRT-seq detected methylation motifs are used as the golden standard.


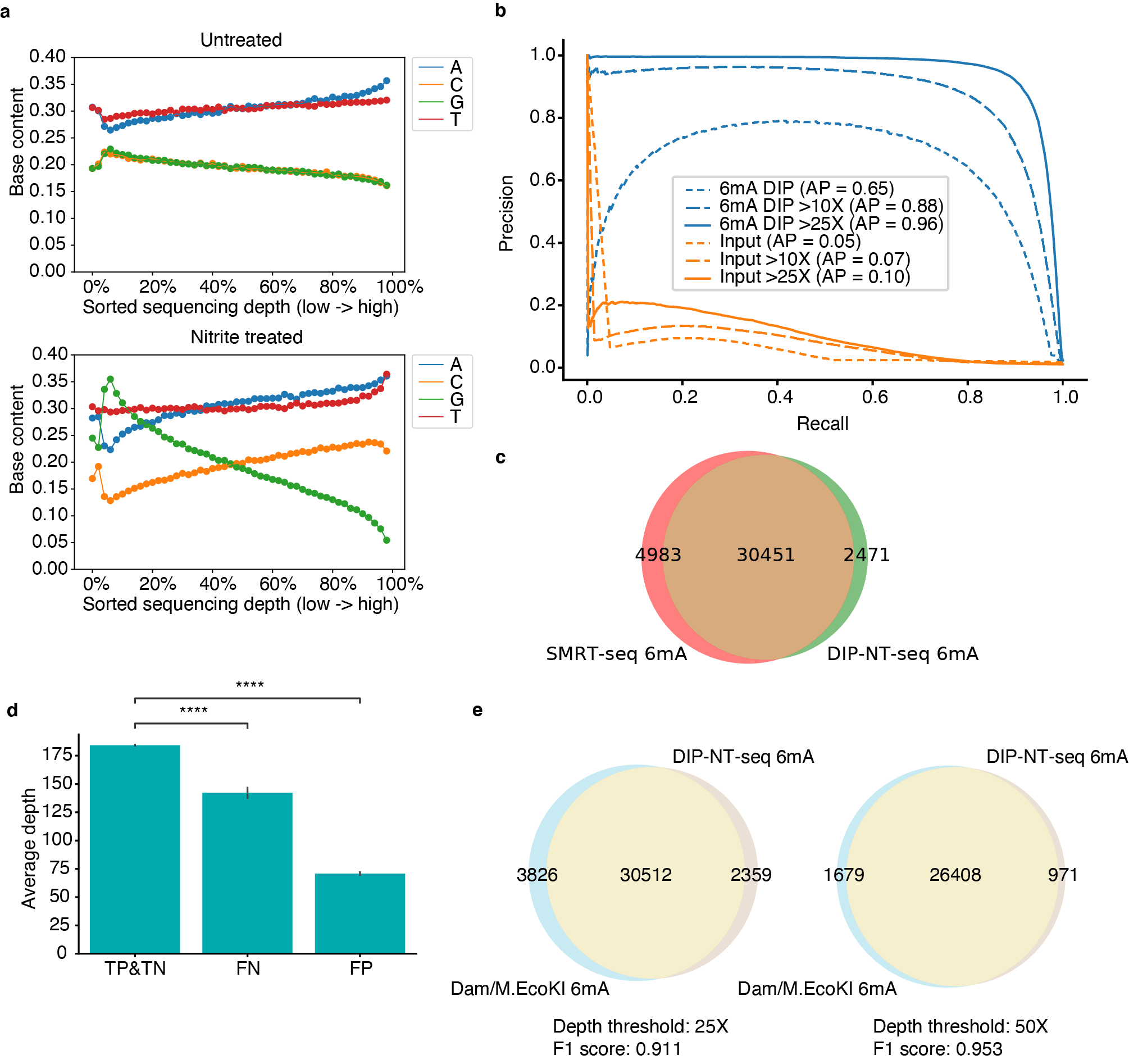


Fig. S18**. Additional information for single-base detection of 6mA by DIP-NT-seq in *E. coli*.** **a**, Correlation between base content and sequencing depth in *H. pylori* genome with or without nitrite treatment. **b**, Precision-Recall curve evaluation of DIP-NT-seq for 6mA detection in *E. coli* genome. **c**, Comparison between SMRT detected 6mA and DIP-NT-seq detected 6mA (Only 6mA sites with sequencing depth >25X in DIP-NT-seq are considered). **d**, Barplot shows that misclassified 6mA positions tend to have low sequencing depth. **e**, Overlap of DIP-NT-seq 6mA and Dam/M.EcoKI 6mA at different sequencing depth thresholds. Statistic tests were performed by two-sided Mann-Whitney-Wilcoxon (MWW) test with Bonferroni correction (****: *P* <= 1.0e-6).


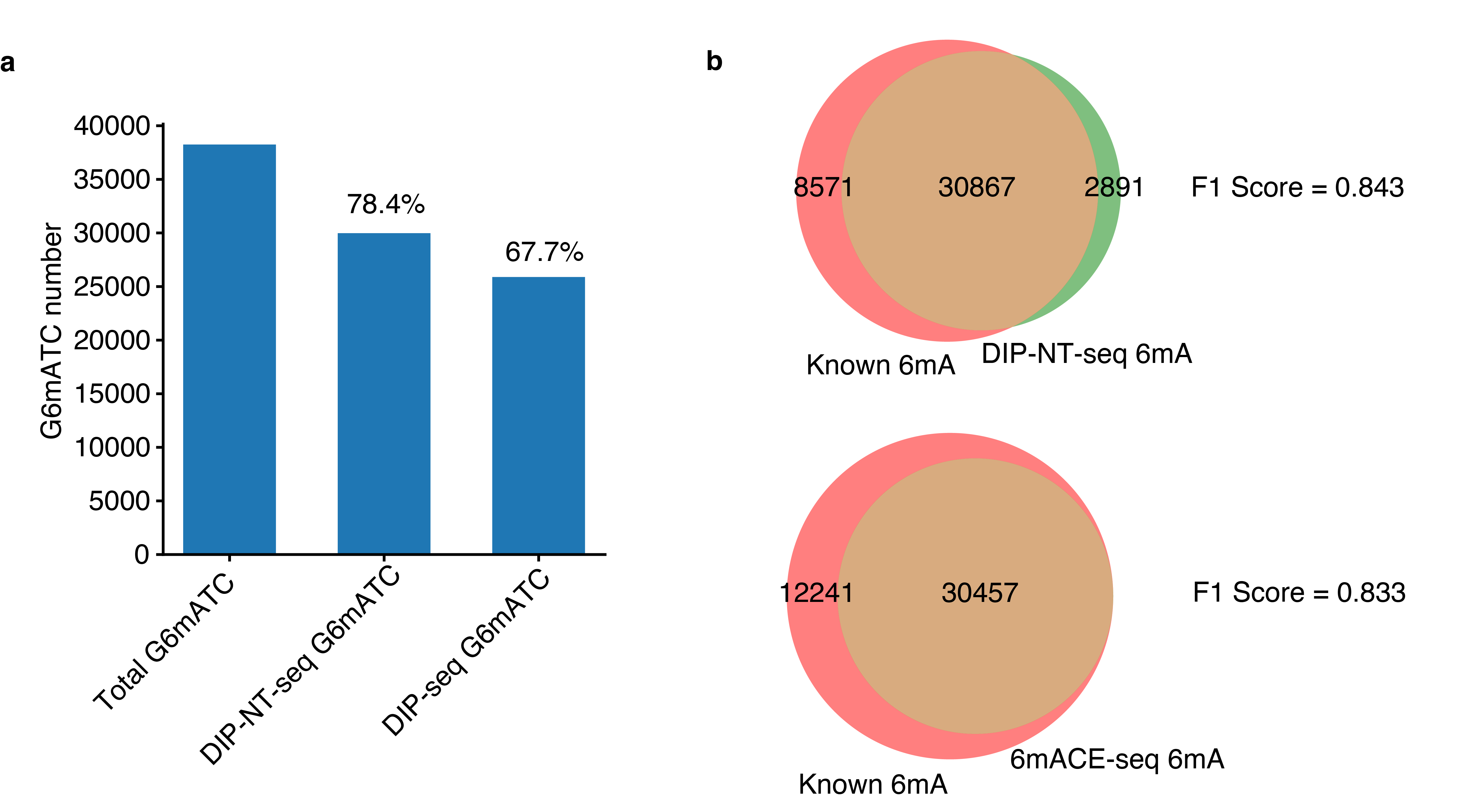


Fig. S19**. Performance comparison between DIP-NT-seq and DIP-seq/6mACE-seq for 6mA detection in *E. coli* genome. a,** Bar chart indicates DIP-NT-seq can detect 11% more 6mA sites within GATC motif in *E. coli* genome than traditional 6mA DIP-seq [18]. **b,** Venn diagrams indicate that DIP-NT-seq (*E. coli* MG1655) detects 6mA at a similar performance as previously reported 6mACE-seq (*E. coli* UTI89) [10] at single-base resolution in the *E. coli* genome.


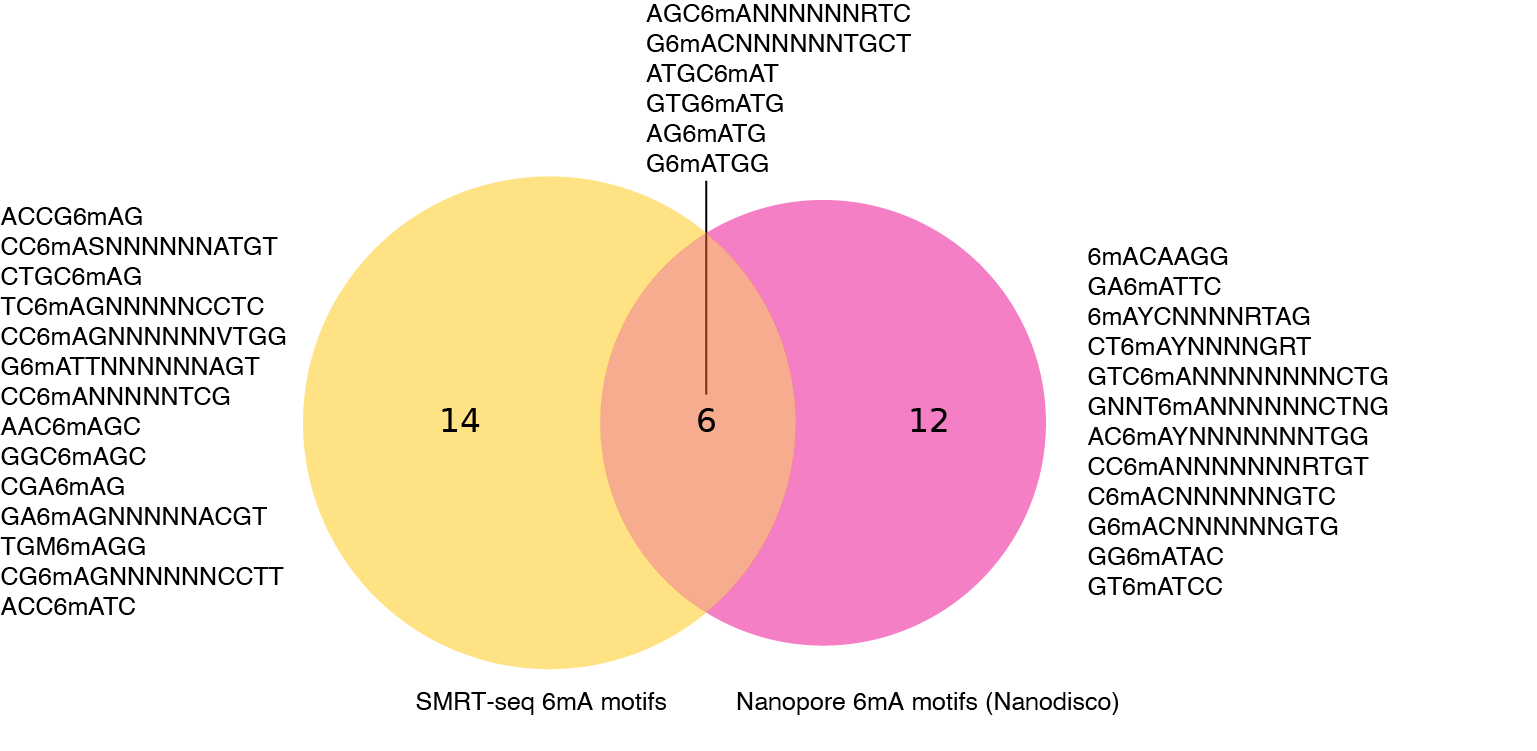


Fig. S20**. Comparison between SMRT-seq detected 6mA motifs and Nanopore sequencing detected 6mA motifs by nanodisco in a mouse gut microbiome sample.**

One-third of the 6mA motifs detected by nanodisco[17] overlap with more reliable SMRT-seq[9] 6mA motifs, indicating current computational tools for Nanopore sequencing are insufficient to reliably detect 6mA motifs from unknown samples.
