# Supplementary material for: NT-seq: a chemical-based sequencing method for genomic methylome profiling
Source: Genome Biol. 2022 May 30;23:122. doi: 10.1186/s13059-022-02689-9 (PMC9150344; doi:10.1186/s13059-022-02689-9)
Supplement: Supplementary file 8 — Additional file 8. Review history. [file 13059_2022_2689_MOESM8_ESM.docx]

Review History

**First round of review**

**Reviewer 1**

**Were you able to assess all statistics in the manuscript, including the appropriateness of statistical tests used?**

Yes.

**Comments to author:**

In the manuscript entitled "NT-Seq: A Chemical-based Sequencing Method for Genomic Methylome Profiling", the authors have developed a novel method called NT-seq (nitrate treatment followed by next-generation sequencing) for mapping genome-wide 6mA, 4mC and 5mC methylation simultaneously. NT-seq expanded the sodium nitrate chemistry which was previously developed on 6mA to 4mC and 5mC. The authors showed that NT-seq could detect all known methylation motifs in model bacteria genome and the results were more accurate and reliable than the third generation sequencing. In addition, the authors have also combined NT-seq with immunoprecipitation to establish DIP-NT-seq for base-resolution sequencing of 6mA. Generally, NT-seq is an interesting method for DNA methylation sequencing, but many problems remain to be solved.

Here are my concerns:

1. In addition to adenine (A) and cytosine (C), guanine (G) can also react with sodium nitrite in acidic solution to generate xanthine (X), which would be read as A in PCR amplification. Therefore, sodium nitrite treatment can induce G-to-A transition on guanine sites. The first question is whether side reaction on guanine will interfere with the detection of 6mA through NT-seq.

2. Lack of characterization of reaction products. For the reaction between 4mC and sodium nitrite, the authors should characterize the potential product (4mC-NO) by LC-MS or NMR to support your hypothesis. Besides, the authors should also analysed the composition of nucleosides by HPLC or LC-MS/MS before and after the sodium nitrate treatment to show the conversion rate of deamination and demonstrate that the reaction would not generate other by-products.

3. The authors demonstrated that NT-seq could quantify 6mA and 5mC frequency in the section "Detection of 6mA, 4mC and 5mC in oligonucleotides using NT-seq". Could NT-seq also quantify 4mC frequency?

4. As is mentioned in the manuscript, the nitrite treatment will cause DNA degradation. Consequently, it is important to know whether the authors optimized the reaction condition on model DNA or ligated DNA by qPCR assay. For ligated DNA, please provide more evidence (for example, agarose gel electrophoresis) on how much DNA could be preserved and whether the DNA would be sheared after sodium nitrate treatment. Additionally, please also compare NT-seq with the antibody-based sequencing for 6mA and bisulfite sequencing for 5mC and 4mC to show which method is more DNA-friendly and requires less DNA input.

5. For 5mC detection, the authors admitted that NT-seq is less efficient than bisulfite sequencing. Since both 5mC and unmodified cytosine would be deaminated during sodium nitrate treatment and their deamination rate has small differences, it is less likely to achieve better performance for 5mC detection by further optimizing the reaction conditions.

6. The authors mentioned that "To explore whether NT-seq can be applied to detect 6mA at single-base resolution, we coupled 6mA DNA immunoprecipitation (6mA-DIP) with NT-seq", which is very confusing. Why the NT-seq itself cannot detect 6mA at single-base resolution? If NT-seq has to be combined with antibody immunoprecipitation to achieve base-resolution sequencing of 6mA, this method would be less useful practically, since there have already been several antibody-based base-resolution sequencing methods for 6mA detection. Moreover, if combined with immunoprecipitation, NT-seq may not be able to detect 6mA, 4mC and 5mC simultaneously and thus the importance of this method would be significantly reduced.

7. It is known that the third generation sequencing is not accurate enough for the detection of DNA methylation, but all the analysis and comparison of methylation motif is between NT-seq and SMRT or Nanopore sequencing. There are several other NGS-based sequencing methods for 6mA and 4mC, such as 6mA-DIP-seq, 6mACE-seq, 6mA-CLIP-exo and 4mC-TAB-seq. The authors should also compare NT-seq with these methods to demonstrate that your method is more sensitive and accurate.

Some small problems:

1. In Figure 1a, the structure of 6mA-NO and 4mC-NO is incorrect.

2. In Figure 4e, "novel 6mA motif" is misspelt.

3. Supplementary Figure 2a should be replaced by a high-resolution version.

**Reviewer 2**

**Were you able to assess all statistics in the manuscript, including the appropriateness of statistical tests used?**

Yes, the authors have used appropriate statistical tests.

**Comments to author:**

This is a nice study from the Dr. Tao Wu's laboratory at Baylor College of Medicine. Three types of DNA methylations, including N6-methyladenine (6mA), N4-methylcytosine (4mC), and C5-methylcytosine (5mC), play essential roles in controlling the genetic information flow. Despite the significance of these modifications, sequencing technologies that enable simultaneously mapping of 6mA, 4mC, and 5mC at a high-resolution genomic scale are still lacking. Recently, a couple of studies showed that the nitrite treatment can be used to detect 6mA, 4mC, and 5mC, however, this treatment has not been adapted to the whole-genome profiling. Dr. Wu et al. demonstrate that NT-seq (nitrite treatment followed by next-generation sequencing) can be used to detect 6mA, 4mC, and 5mC in bacterial genome simultaneously. They show that NT-seq can be used to detect and discover novel methylation motifs. By coupling NT-seq and methyl DNA immunoprecipitation (DIP), NT-seq can profile 6mA at a single-base resolution in both prokaryotes and eukaryotes. Overall, this study presents an innovative method that enable detecting and quantifying DNA methylation at a high-resolution genomic level. The new NT-seq will make significant contributions to the epigenetic field.

Concerns:

1. To this reviewer, it is not easy to follow the experimental design of NT-seq. I would recommend that Figure 1a should be revised by dividing into three groups and incorporating an additional PCR step.

2. The authors have optimized conditions for the nitrite treatment, however, the underlying reasoning is not clearly articulated.

3. The authors have performed the dependence of A to G frequency on percentage of 6mA modified oligo and of C to T frequency on percentage of 5mC, respectively. It is unclear whether a similar liner correlation holds for 4mC.

4. It would be nice if the authors can cross-validate new discovered motifs by other methods.

**Authors Response**

**Point-by-point responses to the reviewers’ comments:**

Reviewer #1:

In the manuscript entitled "NT-Seq: A Chemical-based Sequencing Method for Genomic Methylome Profiling", the authors have developed a novel method called NT-seq (nitrate treatment followed by next-generation sequencing) for mapping genome-wide 6mA, 4mC and 5mC methylation simultaneously. NT-seq expanded the sodium nitrate chemistry which was previously developed on 6mA to 4mC and 5mC. The authors showed that NT-seq could detect all known methylation motifs in model bacteria genome and the results were more accurate and reliable than the third-generation sequencing. In addition, the authors have also combined NT-seq with immunoprecipitation to establish DIP-NT-seq for base-resolution sequencing of 6mA. Generally, NT-seq is an interesting method for DNA methylation sequencing, but many problems remain to be solved.

*Response: We thank the reviewer for the insightful review of our last version manuscript. We conducted several more critical experiments to make this method clear.*

Here are my concerns:

1. In addition to adenine (A) and cytosine (C), guanine (G) can also react with sodium nitrite in acidic solution to generate xanthine (X), which would be read as A in PCR amplification. Therefore, sodium nitrite treatment can induce G-to-A transition on guanine sites. The first question is whether side reaction on guanine will interfere with the detection of 6mA through NT-seq.

*Response: We thank the reviewer for pointing out this common concern of chemical treatment on DNA. First, The NT-seq analysis pipeline we designed can tolerate the G-to-A transition, as we consider A and G the same base during the alignment. Second, Using E. coli whole-genome sequencing data as a mock dataset, after we introduced the G-to-A transition in the FASTQ files to mimic possible nitrite mediated G-to-A transition, the alignment rate indicates that our analysis workflow can align G-to-A converted reads to the reference genome as well as unconverted reads (Supplementary Figure 11b, attached below). Please note here, in our analysis pipeline, we align both converted and unconverted reads to the reference genome. The G-to-A (which aligns to G in reference genome) can be distinguished from 6mA-to-A (which aligns to A in reference genome). Third, we carefully investigated the frequency of G-to-A transition in our NT-seq data of the E. coli genome and found the overall frequency of G-to-A transition is ultra-low (Supplementary Figure 13e, almost all guanine positions are less than 5% transition, also* *attached below). We believe that the frequency of xanthine (X) induced G-to-A transition is dependent on the DNA polymerase (Q5 DNA polymerase was used in the previous study (Mahdavi-Amiri et al. 2021) and Taq DNA polymerase was used in our manuscript).*

*Based on these results, we conclude that the possible G-to-A transition introduced by nitrite treatment will not interface with the detection of adenine and cytosine modification we proposed in the manuscript.*

*Supplementary Figure 11b: Alignment results of E. coli MG1655 strand-specific WGS data (unconverted or artificially introduced AtoG/GtoA/CtoT base change for all FASTQ reads (85bp)) indicate that NT-seq analysis workflow can tolerate all possible base conversions by nitrite treatment.*

*Supplementary Figure 13e: Distribution of NT-seq G to A frequency at G sites in E. coli MG1655 genome from native and PCR amplified DNA*.

2. Lack of characterization of reaction products. For the reaction between 4mC and sodium nitrite, the authors should characterize the potential product (4mC-NO) by LC-MS or NMR to support your hypothesis. Besides, the authors should also analysed the composition of nucleosides by HPLC or LC-MS/MS before and after the sodium nitrate treatment to show the conversion rate of deamination and demonstrate that the reaction would not generate other by-products.

*Response: We thank the reviewer for pointing out this issue. We believe this suggestion can improve the clarity and this should be a critical piece of this method. To validate the proposed reaction from 4mC to 4mC-NO under nitrite treatment, we treated 4mC nucleoside with nitrite condition and performed HPLC-MS/MS experiment before and after the treatment, as the reviewer suggested. The MS results demonstrated that 4mC-NO was indeed formed after nitrite treatment (Supplementary Figure 8c, 4mC-NO [M+Na]+ exact mass: 293.10, found: 293.20, attached below). Similarly, we performed the same nitrite treatment using other unmodified (deoxyadenosine (dA) and deoxycytidine (dC)) and modified (N6-methyl-deoxyadenosine (6mA)) nucleosides. The HPLC trace and MS results indicate there are minimal side products formed after nitrite treatment (Supplementary Figure 1-8). We also analyzed the deamination and nitrosylation rate under our optimized treatment condition using HPLC (Supplementary Figure 9). We found that the nitrosylation of 4mC and 6mA is more rapid than the deamination of dA and dC, which is consistent with the previous report of nitrite treatment on ribonucleosides (adenosine and N6-methyl-adenosine) (Mahdavi-Amiri et al. 2021).*

*Supplementary Figure 8: Mass spectrometry analysis of N4-methyl-deoxycytidine before (a-b) and after (c-d) nitrite treatment.*

3. The authors demonstrated that NT-seq could quantify 6mA and 5mC frequency in the section "Detection of 6mA, 4mC and 5mC in oligonucleotides using NT-seq". Could NT-seq also quantify 4mC frequency?

*Response: We thank the reviewer for raising this question. We performed the NT-seq on oligos with different fractions of 4mC modified oligo and found the C-to-T transition rate is linearly correlated to the percentage of 4mC modified oligo for both positions (r = -0.988 for position 39 and -0.992 for position 42, Figure 2d, 2e, attached below), which is consistent with the results of 6mA and 5mC. These results indicate NT-seq can indeed quantify 4mC frequency with high confidence.*

*Figure 2d, 2e: Linear regression between C to T frequency at 4mC position 39/42 and the percentage of 4mC modified oligo.*

4. As is mentioned in the manuscript, the nitrite treatment will cause DNA degradation. Consequently, it is important to know whether the authors optimized the reaction condition on model DNA or ligated DNA by qPCR assay. For ligated DNA, please provide more evidence (for example, agarose gel electrophoresis) on how much DNA could be preserved and whether the DNA would be sheared after sodium nitrate treatment. Additionally, please also compare NT-seq with the antibody-based sequencing for 6mA and bisulfite sequencing for 5mC and 4mC to show which method is more DNA-friendly and requires less DNA input.

*Response: We thank the reviewer for raising this question. We have optimized the condition using model DNA (156nt DNA oligo) as mentioned in the main text (line 159-169) and eventually determined 2.3% acetic acid, 1M sodium nitrite (pH=4.187), and incubate at 37 ℃ as our condition to move on to genomic DNA. On our hands, this condition balanced the transition efficiency and DNA degradation in the treatment. In terms of degradation level, we performed the nitrite treatment and bisulfite treatment using the same sonicated 293T genomic DNA and found that nitrite treated DNA was less fragmented than the bisulfite treated DNA. However, we agree that the DNA degradation in nitrite treatment is more severe than that in bisulfite treatment (Supplementary Figure 10, attached below). Therefore, it is likely that NT-seq requires more gDNA input than bisulfite sequencing. Compared to the antibody-based sequencing methods for 6mA, 4-5 micrograms of input DNA are commonly used per experiment, we only used 1 microgram of input DNA for NT-seq and DIP-NT-seq in our manuscript with better performance in detecting 6mA.*

*Supplementary Figure 10: DNA degradation comparison between nitrite treatment and bisulfite treatment.*

5. For 5mC detection, the authors admitted that NT-seq is less efficient than bisulfite sequencing. Since both 5mC and unmodified cytosine would be deaminated during sodium nitrate treatment and their deamination rate has small differences, it is less likely to achieve better performance for 5mC detection by further optimizing the reaction conditions.

*Response: We thank the reviewer for pointing this out. Indeed, the difference between 5mC and C is significant but weaker compared to the difference between 4mC or 6mA and their unmodified nucleosides. Therefore, for 5mC, we recommend using NT-seq to detect methylation motifs only in bacterial genomes.*

6. The authors mentioned that "To explore whether NT-seq can be applied to detect 6mA at single-base resolution, we coupled 6mA DNA immunoprecipitation (6mA-DIP) with NT-seq", which is very confusing. Why the NT-seq itself cannot detect 6mA at single-base resolution? If NT-seq has to be combined with antibody immunoprecipitation to achieve base-resolution sequencing of 6mA, this method would be less useful practically, since there have already been several antibody-based base-resolution sequencing methods for 6mA detection. Moreover, if combined with immunoprecipitation, NT-seq may not be able to detect 6mA, 4mC and 5mC simultaneously and thus the importance of this method would be significantly reduced.

*Response: We thank the reviewer for pointing this out. We apologize for the confusing statement here. First, NT-seq itself can detect 6mA and 4mC at single-base resolution with high accuracy (AUC = 0.934 for 6mA and 0.948 for 4mC at positions with more than 25X coverage in the H. pylori genome). The performance of detecting 5mC at single-base resolution is decreased (AUC = 0.832 at positions more than 25X coverage) (Supplementary Figure 17).*

*Second, we indeed find that the performance of 6mA detection can be significantly improved by combining 6mA DNA immunoprecipitation and NT-seq, which we designed to adopt this method to detect the ultra-low level 6mA in mammalian genomes.*

*Thus, we add this part because we think there are a few advantages by combining DNA immunoprecipitation and NT-seq: firstly, it can increase the sensitivity of 6mA detection because of the enrichment and provide a better overall performance in terms of 6mA detection; secondly, it can concentrate the sequencing reads on the 6mA modified region and thus lower the sequencing cost as well as enable the 6mA detection in species with large genomes like Oxytricha and other eukaryotes. Last but not least, this MeDIP-NT-Seq supply a methodology to improve the MeDIP-seq.*

*However, if the reviewer thinks this part will cause the confusion in the audience, we can consider deleting this part from this version.*

7. It is known that the third generation sequencing is not accurate enough for the detection of DNA methylation, but all the analysis and comparison of methylation motif is between NT-seq and SMRT or Nanopore sequencing. There are several other NGS-based sequencing methods for 6mA and 4mC, such as 6mA-DIP-seq, 6mACE-seq, 6mA-CLIP-exo and 4mC-TAB-seq. The authors should also compare NT-seq with these methods to demonstrate that your method is more sensitive and accurate.

*Response: We thank the reviewer for raising this question. Although SMRT and Nanopore sequencing have their own problems, the most available published data are from these two. We have compared our NT-seq results with other NGS-based sequencing methods (Supplementary Figure 19, attached below). We showed that compared to 6mA-DIP-seq, DIP-NT-seq can not only increase the detection resolution to single-base but also detect 11% more GATC sites in the E. coli K-12 genome (Supplementary Figure 19a, attached below). When compared to 6mACE-seq, we found that DIP-NT-seq can detect 7% more 6mA sites but DIP-NT-seq may generate more false-positive sites (Supplementary Figure 19b, attached below). Overall, F1 score indicates that DIP-NT-seq slightly outperformed 6mACE-seq (0.843 for DIP-NT-seq and 0.833 for 6mACE-seq). Additionally, it is worth mentioning that the input material for 6mA-DIP-seq (5ug) and 6mACE-seq (4ug) are much more than our DIP-NT-seq (1ug).*

*Unfortunately, published 6mA-CLIP-exo and 4mC-TAB-seq focus on detecting methylation in species different from those in our manuscript, therefore we are afraid that we cannot directly compare the performance between our method and these two methods.*

*Supplementary Figure 19: Performance comparison between DIP-NT-seq and DIP-seq/6mACE-seq for 6mA detection in E. coli genome.*

Some small problems:

1. In Figure 1a, the structure of 6mA-NO and 4mC-NO is incorrect.

2. In Figure 4e, "novel 6mA motif" is misspelt.

3. Supplementary Figure 2a should be replaced by a high-resolution version.

*Response: For the minor issues, we thank the reviewer for pointing out these. We have corrected the structure of 6mA-NO and 4mC-NO, the misspelled legend in Figure 4e, and replaced the original low-resolution agarose gel image with a high-resolution Tapestation gel image.*

Reviewer #2:

This is a nice study from the Dr. Tao Wu's laboratory at Baylor College of Medicine. Three types of DNA methylations, including N6-methyladenine (6mA), N4-methylcytosine (4mC), and C5-methylcytosine (5mC), play essential roles in controlling the genetic information flow. Despite the significance of these modifications, sequencing technologies that enable simultaneously mapping of 6mA, 4mC, and 5mC at a high-resolution genomic scale are still lacking. Recently, a couple of studies showed that the nitrite treatment can be used to detect 6mA, 4mC, and 5mC, however, this treatment has not been adapted to the whole-genome profiling. Dr. Wu et al. demonstrate that NT-seq (nitrite treatment followed by next-generation sequencing) can be used to detect 6mA, 4mC, and 5mC in bacterial genome simultaneously. They show that NT-seq can be used to detect and discover novel methylation motifs. By coupling NT-seq and methyl DNA immunoprecipitation (DIP), NT-seq can profile 6mA at a single-base resolution in both prokaryotes and eukaryotes. Overall, this study presents an innovative method that enable detecting and quantifying DNA methylation at a high-resolution genomic level. The new NT-seq will make significant contributions to the epigenetic field.

*Response: We thank the reviewer for the positive and insightful review on our work. This encourages us to develop more novel tools for the methylome sequencing.*

Concerns:

1. To this reviewer, it is not easy to follow the experimental design of NT-seq. I would recommend that Figure 1a should be revised by dividing into three groups and incorporating an additional PCR step.

*Response: We thank the reviewer for pointing out this issue. We have added a third column in Figure 1a to show the transformation of the deaminated/nitrosylated base in the PCR step and sequencing step.*

2. The authors have optimized conditions for the nitrite treatment, however, the underlying reasoning is not clearly articulated.

*Response: We thank the reviewer for pointing out this issue. We have added additional description and explanation about the rationale behind the condition optimization (Main text line 159-169 and attached below).*

*“Since the deamination by nitrite treatment is completed through nitrite-mediated diazotization followed by hydrolysis which requires acidic condition and relatively high temperature, the deamination efficiency is positively correlated to acid concentration, incubation temperature, and treatment duration [21]. However, increasing these conditions such as treatment duration can also result in an increased level of DNA degradation (Supplementary Figure 10). Therefore, it is crucial to optimize the treatment condition to achieve a high deamination rate while preserving enough DNA for library preparation when applying it to genomic DNA. Therefore, we treated six pmol (~250ng) protected DNA oligos with different concentrations of acetic acid, different temperatures, and different durations of treatment and performed qPCR to estimate the amount of remaining amplifiable fragments. Considering Ct around 15 as the required amount of amplifiable DNA for downstream library preparation, we determined 2.3% acetic acid, 1M sodium nitrite (pH=4.187), and 37 ℃ treatment as our optimal condition (Supplementary Table 2)”*

3. The authors have performed the dependence of A to G frequency on percentage of 6mA modified oligo and of C to T frequency on percentage of 5mC, respectively. It is unclear whether a similar liner correlation holds for 4mC.

*Response: We thank the reviewer for raising this question. We performed the NT-seq on oligos with a different fraction of 4mC modified oligo and found the C-to-T transition rate is linearly correlated to the percentage of 4mC modified oligo for both positions (r = -0.988 for position 39 and -0.992 for position 42, Figure 2d, 2e, attached below), which is consistent with the results of 6mA and 5mC. These results indicate NT-seq can indeed quantify 4mC frequency with high confidence.*

*Figure 2d, 2e: Linear regression between C to T frequency at 4mC position 39/42 and the percentage of 4mC modified oligo.*

4. It would be nice if the authors can cross-validate new discovered motifs by other methods.

*Response: We thank the reviewer for the suggestion. We have carefully examined whether we can cross-validate the novel motifs we found using NT-seq with independent methods. Fortunately, with published SMRT-seq raw data (Beaulaurier et al. 2018) and 6mA-DIP-seq data (McIntyre et al. 2019), we were able to validate our newly discovered motifs. The detailed information is listed below:*

*1. We found that the previously reported GMRG6mA motif in the H. pylori genome is misclassified because one of the sub-motifs: GAGG6mA is unmodified. This is consistent with SMRT-seq IPD ratio data (Supplementary Figure 14c, attached below).*

*2. Using NT-seq, we also found a novel methylation motif: GGWCN6mA in the H. pylori genome. From the SMRT-seq data, all 8 sub-motifs of GGWCN6mA showed a 1.5-3.5 fold increase in the IPD ratio compared to the background (Supplementary Figure 14d, attached below).*

*3. In the microbial community reference samples, NT-seq detected a novel 6mA motif: AG6mAGG in the L. fermentum genome. Since there is no available SMRT-seq data for this bacteria and no 6mA peak was identified in 6mA-DIP-seq data according to a previous study (McIntyre et al. 2019), we re-analyzed their 6mA-DIP-seq data and were able to identify 1557 6mA peaks by setting macs2 parameter --keep-dup to all. This is likely because the reads “duplication” in the dataset is caused by excessive sequencing depth (15.7 million reads on average for a 1.9 million genome) rather than true PCR duplicates. By further analyzing the identified 1557 6mA peaks, we identified AGAGG as the top significantly enriched* sequence motif in these 6mA peaks, and 75% of AGAGG motifs in the L. fermentum genome overlap with 6mA peaks (Figure 5d, attached below). These data demonstrated the novel 6mA motif: AG6mAGG detected by our NT-seq is a true 6mA motif in the L. fermentum genome.

*Reference:*

*Beaulaurier J, Zhu S, Deikus G, et al (2018) Metagenomic binning and association of plasmids with bacterial host genomes using DNA methylation. Nat Biotechnol 36:61–69. https://doi.org/10.1038/nbt.4037.*

*Mahdavi-Amiri Y, Chung Kim Chung K, Hili R (2021) Single-nucleotide resolution of: N 6-adenine methylation sites in DNA and RNA by nitrite sequencing. Chem Sci 12:606–612. https://doi.org/10.1039/d0sc03509b.*

*McIntyre ABR, Alexander N, Grigorev K, et al (2019) Single-molecule sequencing detection of N6-methyladenine in microbial reference materials. Nat Commun 10:579. https://doi.org/10.1038/s41467-019-08289-9.*

**Second round of review**

**Reviewer 1**

The authors have addressed my concerns in the revision.

**Reviewer 2**

Dr. Wu has thoroughly addressed my comments. I recommend to publish this important piece of work.
